# Supplementary material for: Histone acetylation homeodynamics navigates cell survival and apoptosis
Source: Nat Commun. 2025 Dec 12;16:11358. doi: 10.1038/s41467-025-66405-4 (PMC12728225; doi:10.1038/s41467-025-66405-4)
Supplement: Supplementary file 1 — Supplementary Information [file 41467_2025_66405_MOESM1_ESM.pdf]

Supplementary information

Histone acetylation homeodynamics navigates cell survival and apoptosis

Kang Li,[1,2,#] Ling Tian,[3,#], Wenxin Cao,[1,#], Jiahui Zhang,[1] Wenhao Zhang,[4] Tong Lin,[1] Shumin Huang,[1] Yongyu Qiu,[1] Zifeng Ruan,[5] Jianhao Deng,[3] Shihui Long,[1] Subba R. Palli,[6] and Sheng Li[1,2,*]

[1]Guangdong Provincial Key Laboratory of Insect Developmental Biology and Applied Technology, Institute of Insect Science and Technology, School of Life Sciences, South China Normal University, Guangzhou, China
[2]Guangmeiyuan R&D Center, Guangdong Provincial Key Laboratory of Insect Developmental Biology and Applied Technology, South China Normal University, Meizhou, Meizhou, China
[3]Guangdong Provincial Key Laboratory of Agro-animal Genomics and Molecular Breeding/Guangdong Provincial Sericulture and Mulberry Engineering Research Center, College of Animal Science, South China Agricultural University, Guangzhou, China
[4]College of Biological and Food Engineering, Huaihua University, Huaihua, China
[5]CAS Key Laboratory of Regenerative Biology, Joint School of Life Sciences, Guangzhou Institutes of Biomedicine and Health, Chinese Academy of Sciences; University of Chinese Academy of Sciences, Beijing, China
[6]Department of Entomology, College of Agriculture, Food, and Environment, University of Kentucky, Lexington, USA
[#]Equal contribution
*Correspondence: lisheng@scnu.edu.cn (S.L.)

This file contains Supplementary Figures 1-16 and Supplementary table 1-6

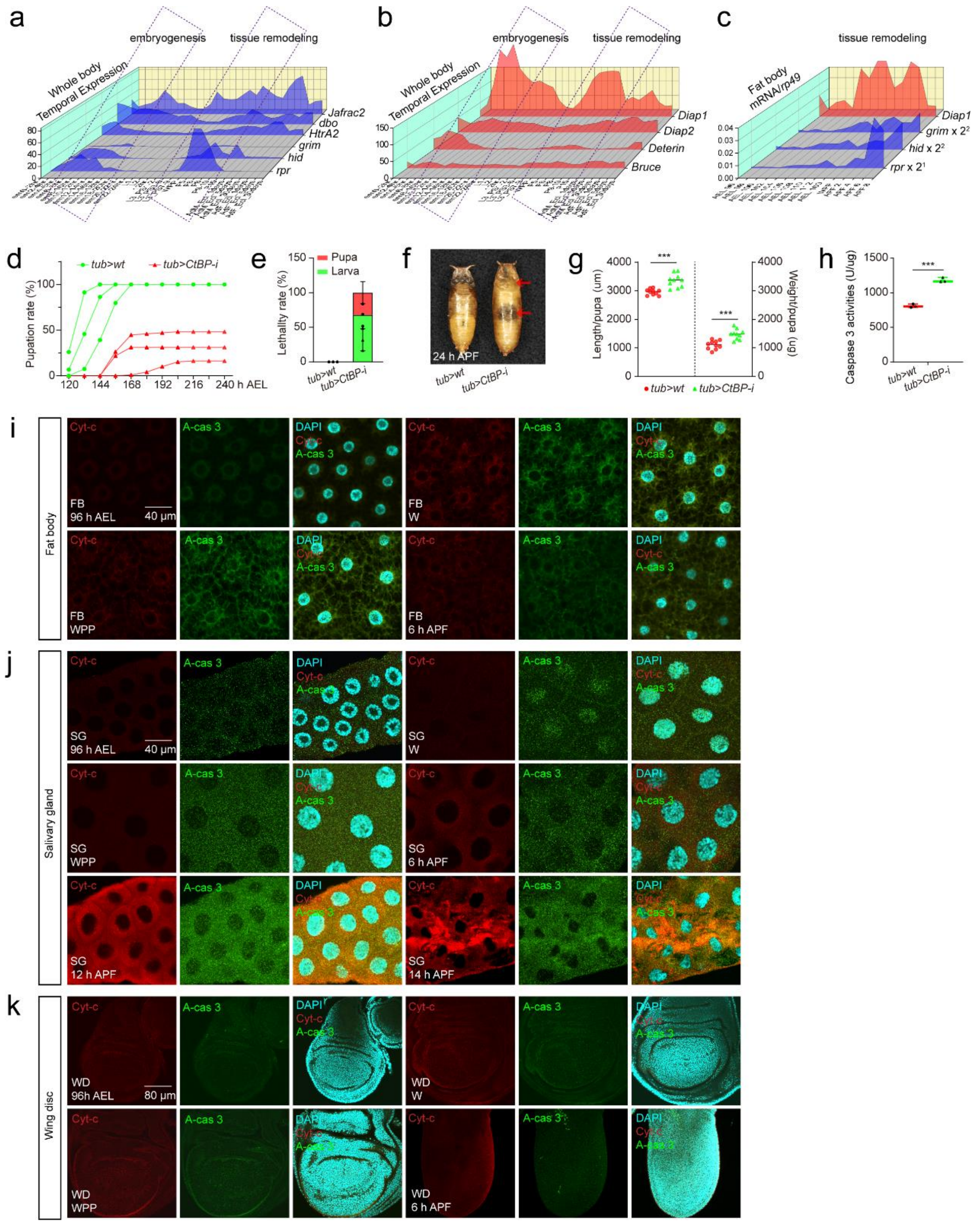

Supplementary Figure 1. The balance between PAPs and IAPs, the phenotype after global *CtBP-i*, and apoptosis detection in three tissues during the larval–pupal transition, related to introduction and Figure 1

(**a** and **b**) RPKM values showing the global developmental profiles of genes encoding *PAPs* (**a**) and *IAPs* (**b**) in *Drosophila*, RPKM data were obtained from FlyBase.

(**c**) Relative transcriptional profiles (average of three repeated experiments) of genes encoding *PAPs* and *IAPs* in the fat body from 96 h AEL to 8 h APF, and *rp49* was used

as the reference gene. To enhance the visual representation of gene expression dynamics, the Z-axis values for selected genes were amplified by a factor of 2^n. AEL, after egg laying. APF, after puparium formation.

(**d**-**h**) Global *CtBP-i* with *tub-Gal4*, *tub-Gal4>wt* was used as control. Developmental time statistics (**d**), lethality statistics (**e**), phenotype observation at pupal stage (**f**), body volume and weight (**g**), n = 10 independent pupa from control and RNAi group. Caspase 3 activity measurement in the whole body (**h**), 5 lavar as one group. In **e** and **h**, mean ± SD; n = 3 independent samples. In **g** and **h**, two-tailed paired *t* test: ***p <0.001.

(**i**-**k**) Developmental profiles of Cyt-c and active caspase 3 (A-cas 3) detected by immunofluorescence staining in the fat body (96 h AEL-6 h APF) (**i**), salivary glands (96 h AEL-14 h APF) (**j**), and wing discs (96 h AEL-6 h APF) (**k**) during the larval–pupal transition.

Data in **f** is representative of three independent experiments with similar results. Source data are provided as a Source Data file. The genotypes are provided in Supplementary Table 3.

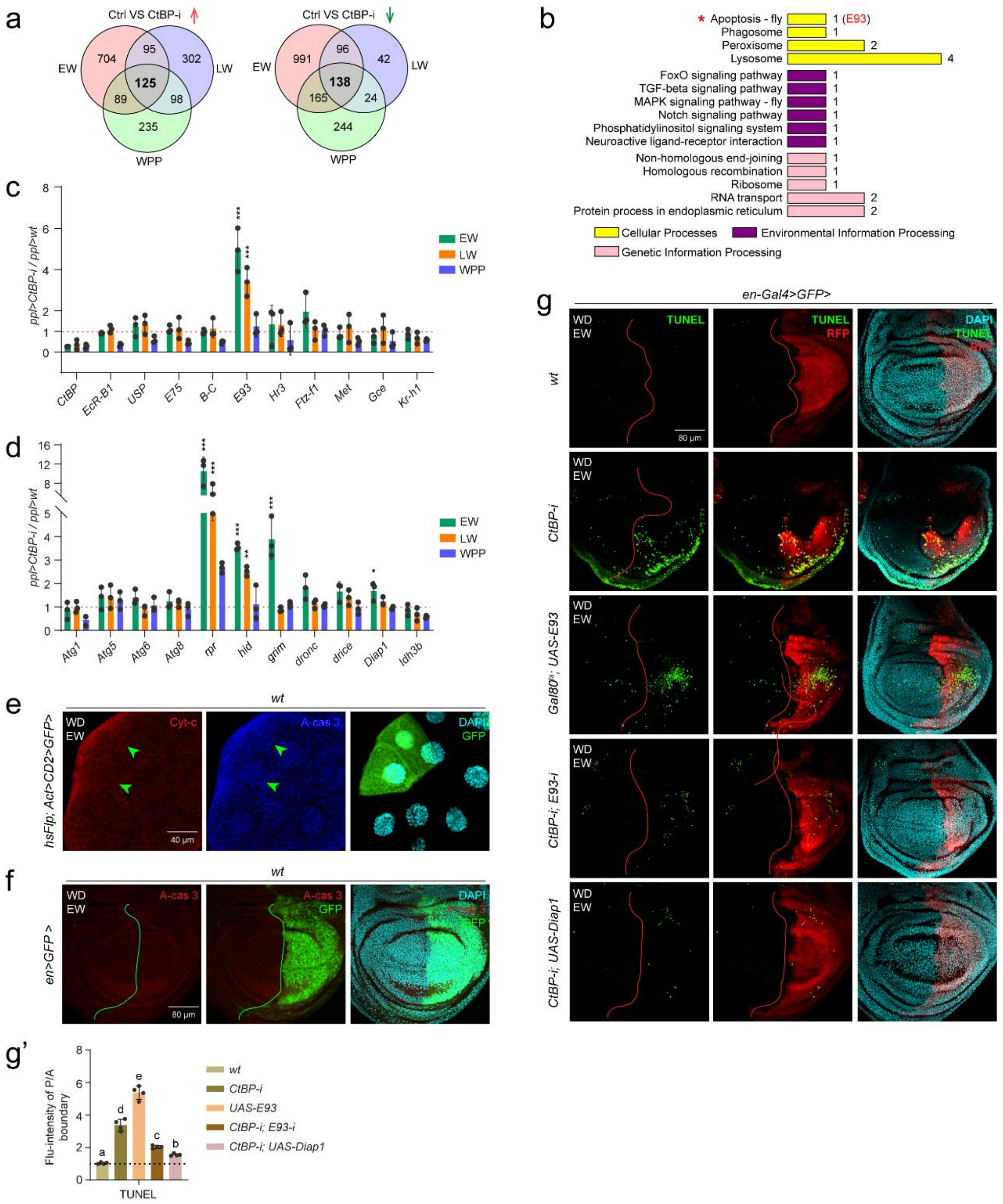

Supplementary Figure 2. Downregulation of *CtBP* induces *E93* expression and disrupts the balance between *PAPs* and *IAPs*, related to Figure 1

(**a** and **b**) Fat body-specific *CtBP-i* results in gene expression changes at three developmental stages detected by RNA-seq. Venn diagram showing the numbers of up- and downregulated genes (**a**), up indicated by red arrow: log_2FoldChange > 0.58; down indicated by green arrow: log_2FoldChange < -0.58. KEGG pathway enrichment analysis of upregulated genes (**b**). EW, early wandering; LW, late wandering; WPP, white prepupae.

(**c** and **d**) Relative transcript levels of the 20E (*EcR-B1*, *USP*, *E75*, *Br-C*, *E93*, Hr3, *Ftz-f1*) and JH (*Met*, *Gce*, and *Kr-h1*) signaling transduction genes (**c**); autophagy-related genes (*Atg1*, *Atg5*, *Atg6*, and *Atg8*), and genes encoding *PAPs* (*rpr*, *hid*, and *grim*), *IAPs* (*Diap1*), *caspase* (*dronc* and *drice*), and *Idh3b* (involved in autophagic cell death) (**d**) in the fat body after *CtBP-i* using *ppl-Gal4*, *ppl-Gal4>wt* was used as control. Mean ± SD; n = 3 independent samples. Two-tailed paired *t* test: **p* <0.05, ***p* <0.01, ****p* <0.001.

(**e**) The immunofluorescence (IF) staining of active caspase 3 (A-cas 3) and Cyt-c in the negative control of *hsFlp; Act>CD2>GFP>wt* in the salivary glands at EW stage. Both GFP clone and none GFP clone are *wt* cells.

(**f**) The IF of A-cas 3 in the negative control of *en-Gal4>wt* in the wing disc at EW stage. Both the posterior (P) boundaries indicated by GFP and the anterior (A) boundaries are *wt* cells.

(**g** and **g'**) After *CtBP-i*, *UAS-E93*, *CtBP-i* & *E93-i*, and *CtBP-i* & *UAS-Diap1* at the EW, TUNEL staining was performed to evaluate apoptosis in the wing disc using *en-Gal4* or *Gal80^{ts}, en-Gal4* line (**g**). The fluorescence intensity of TUNEL in the P boundary compared to the A boundary in the wing disc (**g'**), mean ± SD; n = 4 independent wing disc. One-way ANOVA: different lowercase letters are significantly different ($P < 0.05$).

Source data are provided as a Source Data file. The genotypes are provided in Supplementary Table 3.

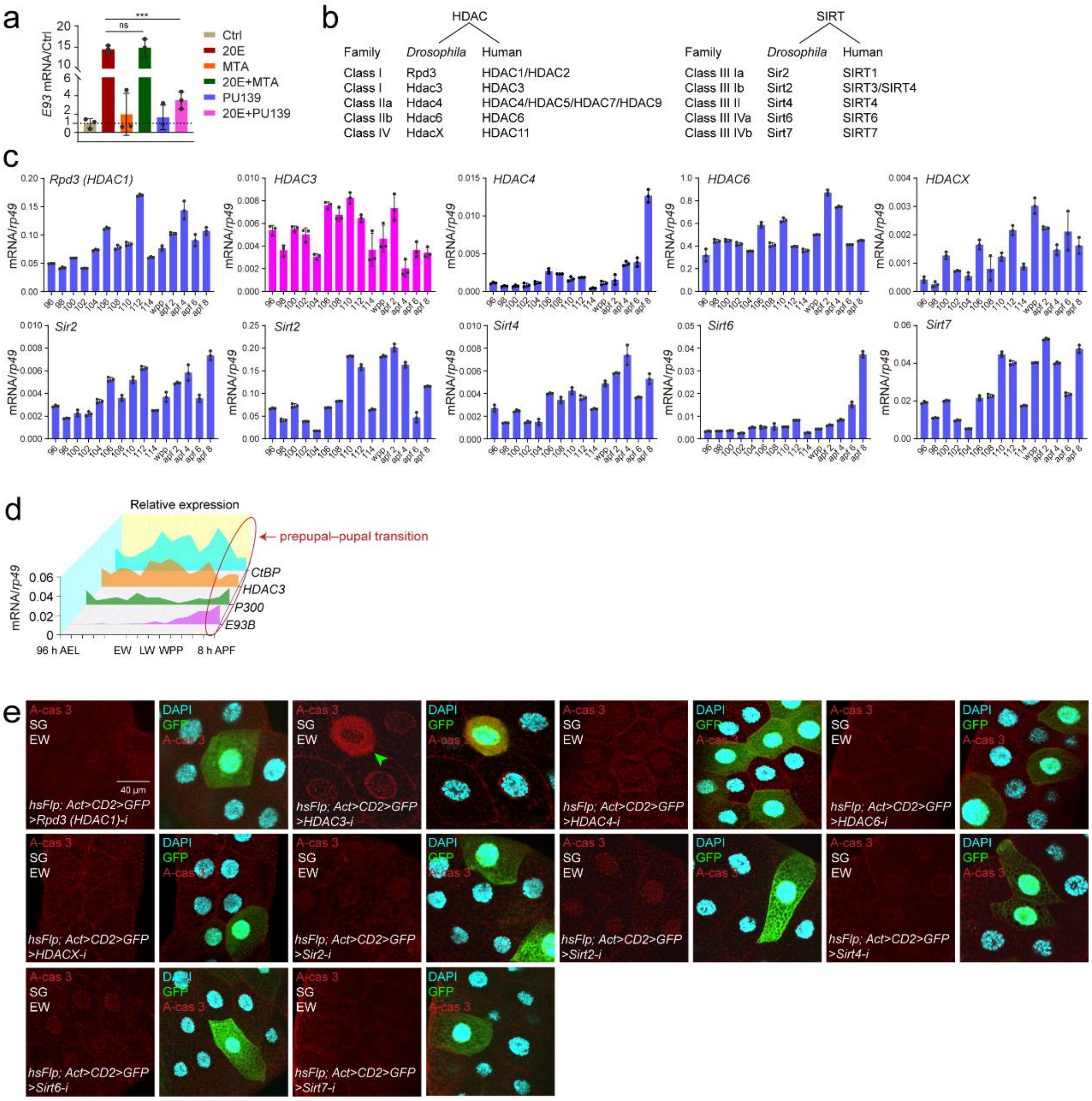

Supplementary Figure 3. Chemical treatment and RNAi screening to identify the suppression of apoptosis by HDAC3, related to Figure 2

(**a**) Pretreated with MTA and PU139 for 12 h, measurement of *E93* mRNA expression after 20E treatment for 4 h in Kc cells. Mean ± SD; n = 3 independent samples. Two-tailed paired *t* test: ***p <0.001. ns, not significant.

(**b**) Homology of HDACs in *Drosophila* and mammals.

(**c**) Transcriptional profiles of ten *HDACs* in the fat body from 96 h AEL to 8 h APF. Mean ± SEM. n = 3 independent samples.

(**d**) Relative transcriptional profiles (average of three repeated experiments) of *CtBP*, *HDAC3*, *P300* and *E93* in the fat body from 96 h AEL to 8 h APF, and *rp49* was used as the reference gene. AEL, after egg laying; APF, after puparium formation.

(**e**) Identification of HDACs that regulate apoptosis using Flp-out-mediated RNA-i in the salivary glands at the EW. GFP indicates clone cells with genes RNA-i. The green arrow represents cells undergoing apoptosis detected by the immunofluorescence of A-cas 3.

Source data are provided as a Source Data file. The genotypes are provided in Supplementary Table 3.

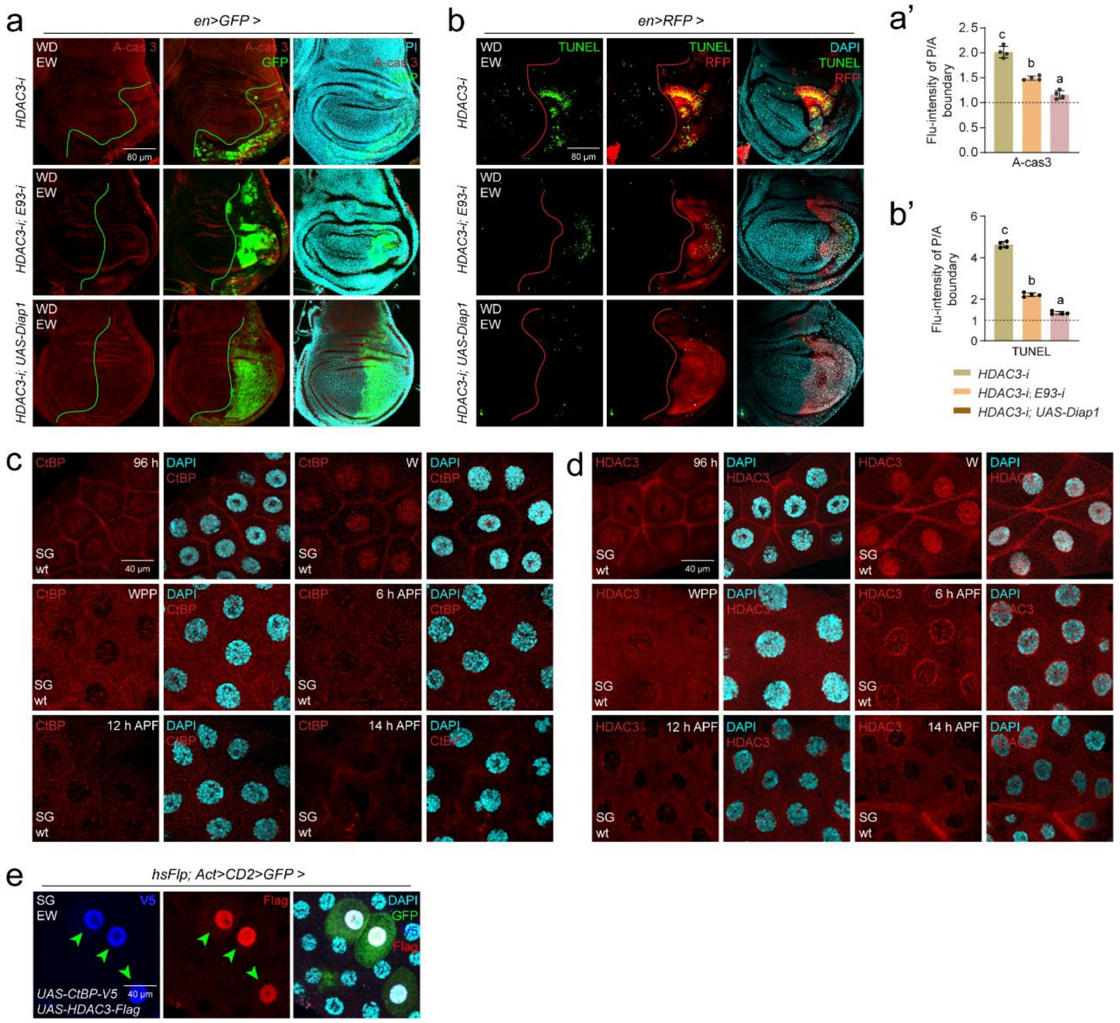

Supplementary Figure 4. CtBP-HDAC3 suppresses the expression of *E93* and *PAPs*, related to Figure 2

(**a** and **a'**) After *HDAC3-i*, *HDAC3-i* & *E93-i*, and *HDAC3-i* & *UAS-Diap1* at the EW, immunofluorescence (IF) staining was performed to evaluate A-cas 3 in the wing discs using *en-Gal4* line (a). GFP region indicates the posterior (P) boundaries of the wing disc where gene RNA-i or overexpression is manipulated. The fluorescence intensity of A-cas 3 in the P boundary compared to the anterior (A) boundary in the wing disc in **a** (**a'**). Mean ± SD; n = 4 independent wing disc. One-way ANOVA: different lowercase letters are significantly different ($P < 0.05$).

(**b** and **b'**) After *HDAC3-i*, *HDAC3-i* & *E93-i*, and *HDAC3-i* & *UAS-Diap1* at the EW, TUNEL staining was performed to evaluate apoptosis in the wing disc using *en-Gal4* line (**b**). The fluorescence intensity of TUNEL in the P boundary compared to the A boundary in the wing disc in **b** (**b'**). Mean ± SD; n = 4 independent wing disc. One-way

ANOVA: different lowercase letters are significantly different ($P < 0.05$).

(**c** and **d**) Developmental profiles and cell location of CtBP (**c**) and HDAC3 (**d**) protein detected by IF staining in the salivary glands from 96 h AEL to 14 h APF.

(**e**) IF staining of V5 and Flag to show the colocalization of CtBP-V5 and HDAC3-Flag in the salivary glands nuclei at the EW using Flp-out line. GFP clone cells represent CtBP-V5 and HDAC3-Flag overexpression clone cells.

Source data are provided as a Source Data file. The genotypes are provided in Supplementary Table 3.

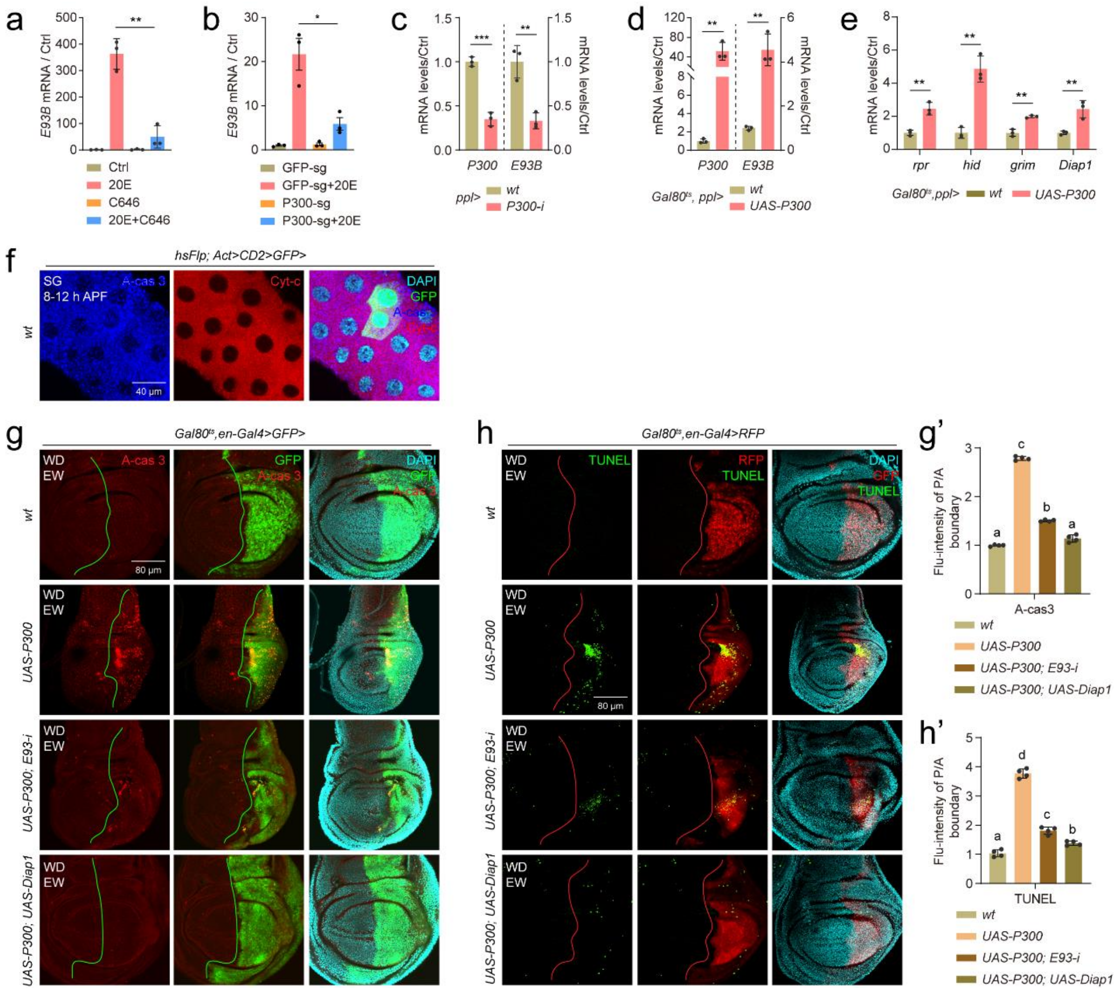

Supplementary Figure 5. Chemical treatment and functional verification of P300 regulating apoptosis, related to Figure 3

(**a**) Transcript levels of *E93B* in Kc cells after treatment with 20E, C646, or both for 12 h. Mean ± SD; n = 3 independent samples. Two-tailed paired *t* test: $^{**}p<0.01$.

(**b**) Transcript level of *E93B* in Kc cells after knockdown *P300* or/and 20E treatment for 4 h. Mean ± SD; n = 3 independent samples. Two-tailed paired *t* test: $^{*}p<0.05$.

(**c**) Transcript levels of *P300* and *E93B* after *P300-i* at 6 h APF in the fat body using *ppl-Gal4*, *ppl-Gal4>wt* was used as control. Mean ± SD; n = 3 independent samples. Two-tailed paired *t* test: $^{**}p<0.01$, $^{***}p<0.001$.

(**d** and **e**) Transcript levels of *P300* and *E93B* (**d**), and *PAPs* and *IAPs* (**e**) after *UAS-P300* at the EW in the fat body using *Gal80*^{ts}*; ppl-Gal4*, *Gal80*^{ts}*; ppl-Gal4>wt* was used as control. Mean ± SD; n = 3 independent samples. Two-tailed paired *t* test: $^{**}p<0.01$,$^{***}p<0.001$.

(**f**) The immunofluorescence of A-cas 3 and Cyt-c in the negative control of *hsFlp; Act>CD2>GFP>wt* in the salivary glands at 8-12 h APF. Both GFP clone and none GFP clone are *wt* cells.

(**g** and **g'**) After *UAS-P300*, *UAS-P300 & E93-i*, and *UAS-P300 & UAS-Diap1*, IF staining of A-cas3 in the wing discs using *Gal80^{ts}, en-Gal4* line at the EW (**g**). The fluorescence intensity of A cas 3 in the P boundary compared to the A boundary (**g'**). Mean ± SD; n = 4 independent wing disc. One-way ANOVA: different lowercase letters are significantly different ($P < 0.05$).

(**h** and **h'**) After *UAS-P300*, *UAS-P300 & E93-i*, and *UAS-P300 & UAS-Diap1*, IF staining of TUNEL in the wing discs using *Gal80^{ts}, en-Gal4* line at the EW (**h**). The fluorescence intensity of TUNEL in the P boundary compared to the A boundary (**h'**). Mean ± SD; n = 4 independent wing disc. One-way ANOVA: different lowercase letters are significantly different ($P < 0.05$).

Source data are provided as a Source Data file. The genotypes are provided in Supplementary Table 3.

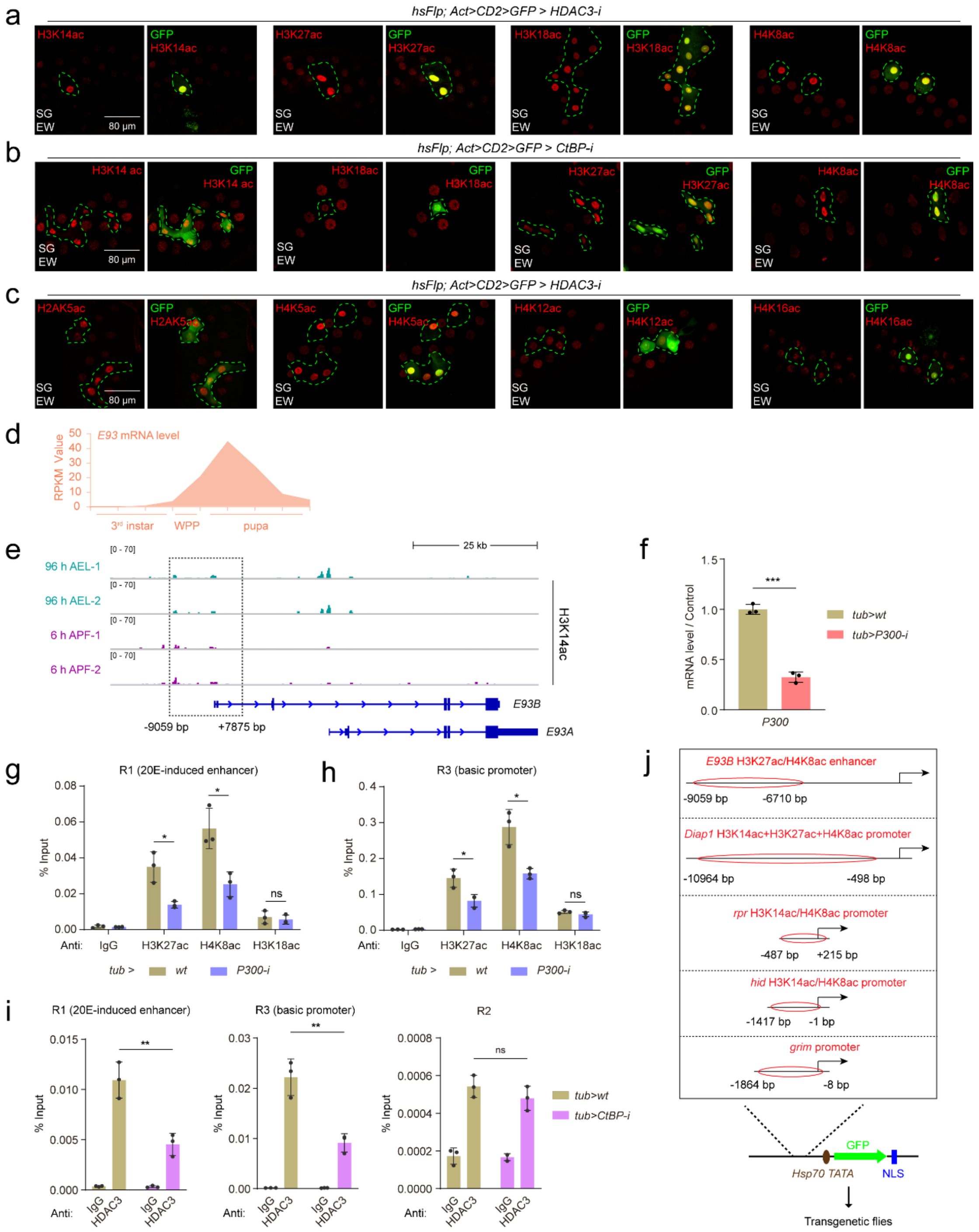

Supplementary Figure 6. P300-CtBP/HDAC3 modulates H3K27ac/H4K8ac homeodynamics in the *E93* enhancer and promoter, related to Figure 3

(**a**) Evaluation of H3K14ac, H3K18ac, H3K27ac, and H4K8ac after *HDAC3-i* in the salivary glands using Flp-out line at the EW. GFP clone cells indicate *HDAC3-i* cells.

(**b**) Evaluation of H3K14ac, H3K18ac, H3K27ac, and H4K8ac after *CtBP-i* in the salivary glands using Flp-out line at the EW. GFP clone cells indicate *CtBP-i* cells.

(**c**) Evaluation of H2AK5ac, H4K5ac, H4K12ac, and H4K16ac after *HDAC3-i* in the

salivary glands using Flp-out line at the EW. GFP clone cells indicate *HDAC3-i* cells.

(**d**) The orange area chart represents global development pattern of *E93* transcription. The RPKM data were obtained from FlyBase.

(**e**) The Integrative Genomics Viewer (IGV) tracks showing global H3K14ac at the *E93* gene locus at 96 h AEL and 6 h APF.

(**f**) Transcript levels of *P300* after global *P300-i* at 6 h APF using *tub-Gal4*, *tub-Gal4>wt* was used as control. Mean ± SD; n = 3 independent samples. Two-tailed paired *t* test: ***p <0.001.

(**g** and **h**) Enrichment of H3K27ac, H4K8ac, and H3K14ac in the 20E-induced enhancer-R1 (**g**) and basic promoter-R3 (**h**) detected by ChIP-qPCR, after global *P300-i* at 6 h APF using *tub-Gal4*, *tub-Gal4>wt* was used as control. Primers for ChIP-qPCR were located in R1 and R3. Mean ± SD; n = 3 independent samples. Two-tailed paired *t* test: *p <0.05. ns, not significant.

(**i**) DNA enrichment of HDAC3 in the 20E-induced enhancer-R1, basic promoter-R3, and R2 (negative control) detected by ChIP-qPCR, after global *CtBP-i* at the EW using *tub-Gal4*, *tub-Gal4>wt* was used as control. Primers for ChIP-qPCR were located in R1, R2, and R3. Mean ± SD; n = 3 independent samples. Two-tailed paired *t* test: **p <0.01. ns, not significant.

(**j**) Diagram showing gene enhancer-GFP or promoter-GFP for transgenic flies. *Hsp70 TATA*, *Hsp70 TATA* mini promoter. NLS, nuclear localization signal. The red ellipse represents the enhancer or promoter region that undergoes histone modification, which was then constructed into the pH-Stinger vector and used to prepare transgenic fruit flies.

Source data are provided as a Source Data file. The genotypes are provided in Supplementary Table 3.

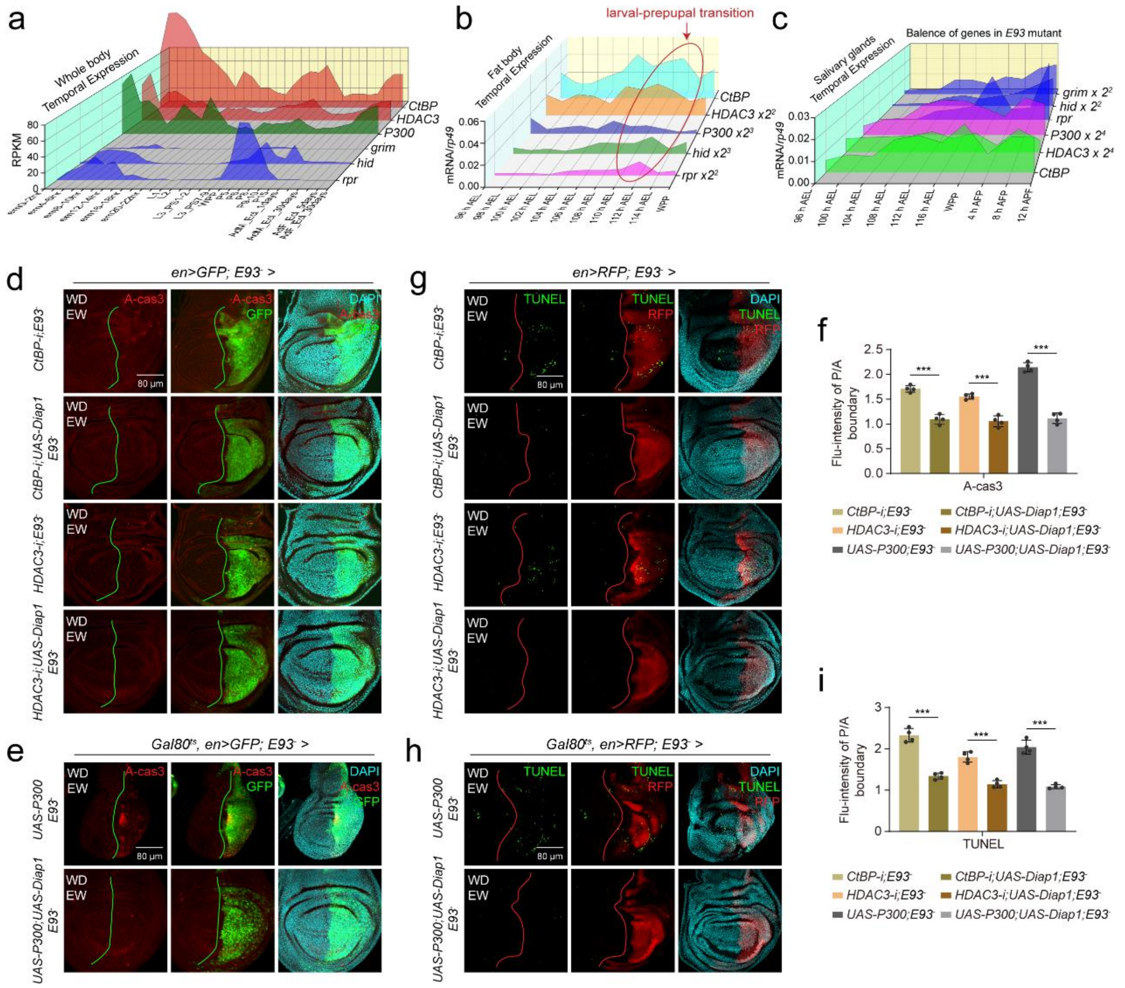

Supplementary Figure 7. Detection of P300-CtBP/HDAC3 regulating *PAPs* in *E93* mutant, related to Figure 4

(**a**) RPKM values showing the global transcriptional profiles of *CtBP*, *HDAC3*, *P300*, and *PAPs* in *Drosophila*. The RPKM data were obtained from FlyBase.

(**b**) Relative transcriptional profiles (average of three repeated experiments) of *CtBP*, *HDAC3*, *P300*, and *PAPs* (*rpr*/*hid*) in the fat body from 96 h AEL to WPP. *Rp49* was used as the reference gene. To enhance the visual representation of gene expression dynamics, the Z-axis values for selected genes were amplified by a factor of 2^n.

(**c**) Relative transcriptional profiles (average of three repeated experiments) of *CtBP*, *HDAC3*, *P300*, and *PAPs* (*rpr*/*hid*/*grim*) in *E93* mutant salivary glands from 96 h AEL to 12 h APF. *Rp49* was used as the reference gene. To enhance the visual representation of gene expression dynamics, the Z-axis values for selected genes were amplified by a factor of 2^n.

(**d**-**f**) In the *E93* mutant background, after *CtBP-i*, *CtBP-i* & *UAS-Diap1*, *HDAC3-i*, *HDAC3-i* & *UAS-Diap1* using *en-Gal4* (**d**), *UAS-P300* and *UAS-P300* & *UAS-Diap1* using *Gal80*^{ts}*; en-Gal4* (**e**) at the EW, IF staining was performed to evaluate A-cas 3 in the wing disc. The fluorescence intensity of A-cas 3 in the P boundary compared to the A boundary in **d** and **e** (**f**). Mean ± SD; n = 4 independent wing disc. Two-tailed paired *t* test: ***p <0.001.

(**g**-**i**) In the *E93* mutant background, after *CtBP-i*, *CtBP-i* & *UAS-Diap1*, *HDAC3-i*, *HDAC3-i* & *UAS-Diap1* using *en-Gal4* (**g**), *UAS-P300* and *UAS-P300* & *UAS-Diap1* using *Gal80*^{ts}*; en-Gal4* (**h**) at the EW, TUNEL staining was performed to evaluate apoptosis in the wing disc. The fluorescence intensity of TUNEL in the P boundary compared to the A boundary in **g** and **h** (**i**). Mean ± SD; n = 4 independent wing disc. Two-tailed paired *t* test: ***p <0.001.

Source data are provided as a Source Data file. The genotypes are provided in Supplementary Table 3.

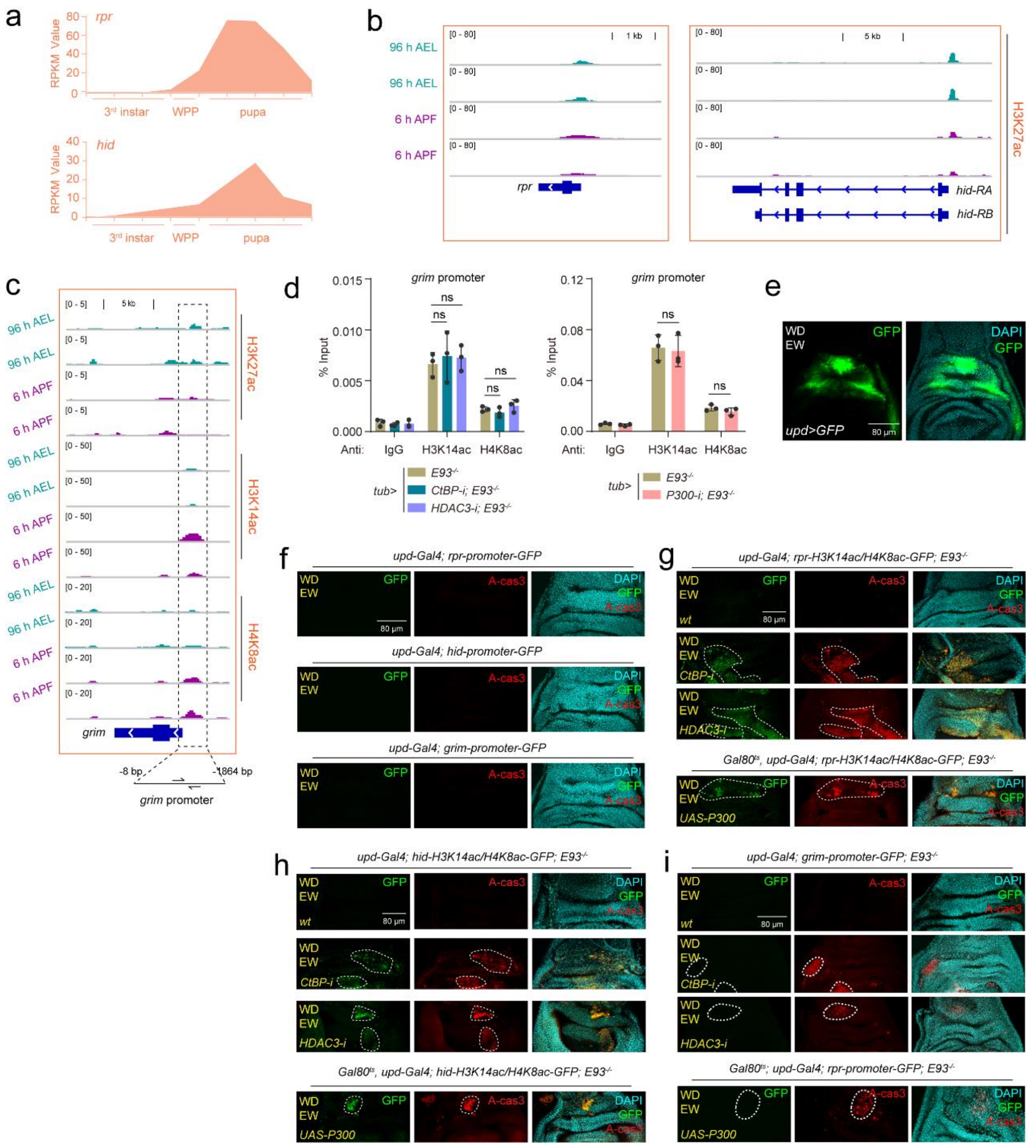

Supplementary Figure 8. P300-CtBP/HDAC3 modulates H3K14ac/H4K8ac homeodynamics in the *rpr* and *hid* promoters, related to Figure 4

(**a**) The orange area chart represents global development pattern of *rpr*/*hid* transcription. The RPKM data were obtained from FlyBase.

(**b**) IGV tracks showing global H3K27ac at the *rpr* and *hid* gene loci at 96 h AEL and 6 h APF.

(**c**) IGV tracks showing global H3K14ac/H3K27ac/H4K8ac at the *grim* gene locus at 96 h AEL and 6 h APF.

(**d**) Enrichment of H3K14ac and H4K8ac in the *grim* promoter detected by ChIP-qPCR,

after global *CtBP-i*, *HDAC3-i*, and *P300-i* in the *E93* mutant at 6 h APF using *tub-Gal4*. Primers for ChIP-qPCR were located in *grim* promoter. Mean ± SD; n = 3 independent samples. Two-tailed paired *t* test: ns, not significant.

(**e**) Location of GFP expression using *upd-Gal4*.

(**f**) Detection of the GFP signal indicating H3K14ac/H4K8ac promoters of *rpr*, *hid*, and *grim* promoter in *wt* background in the wing disc at the EW using *upd-Gal4*.

(**g** and **h**) Detection of the GFP signal indicating H3K14ac/H4K8ac level in the *rpr* (**g**) and *hid* (**h**) promoters after *CtBP-i*, *HDAC3-i*, and *UAS-P300* in the wing discs using *upd-Gal4* or *Gal80*^{ts}*; upd-Gal4* at the EW. IF of A-cas 3 (red fluorescence) was used to indicate cells occur apoptosis.

(**i**) Detection of the GFP signal indicating the *grim* promoter after *CtBP-i*, *HDAC3-i*, and *UAS-P300* in the wing disc at the EW using *upd-Gal4* or *Gal80*^{ts}*; upd-Gal4*. IF of A-cas 3 (red fluorescence) was used to indicate cells occur apoptosis.

Source data are provided as a Source Data file. The genotypes are provided in Supplementary Table 3.

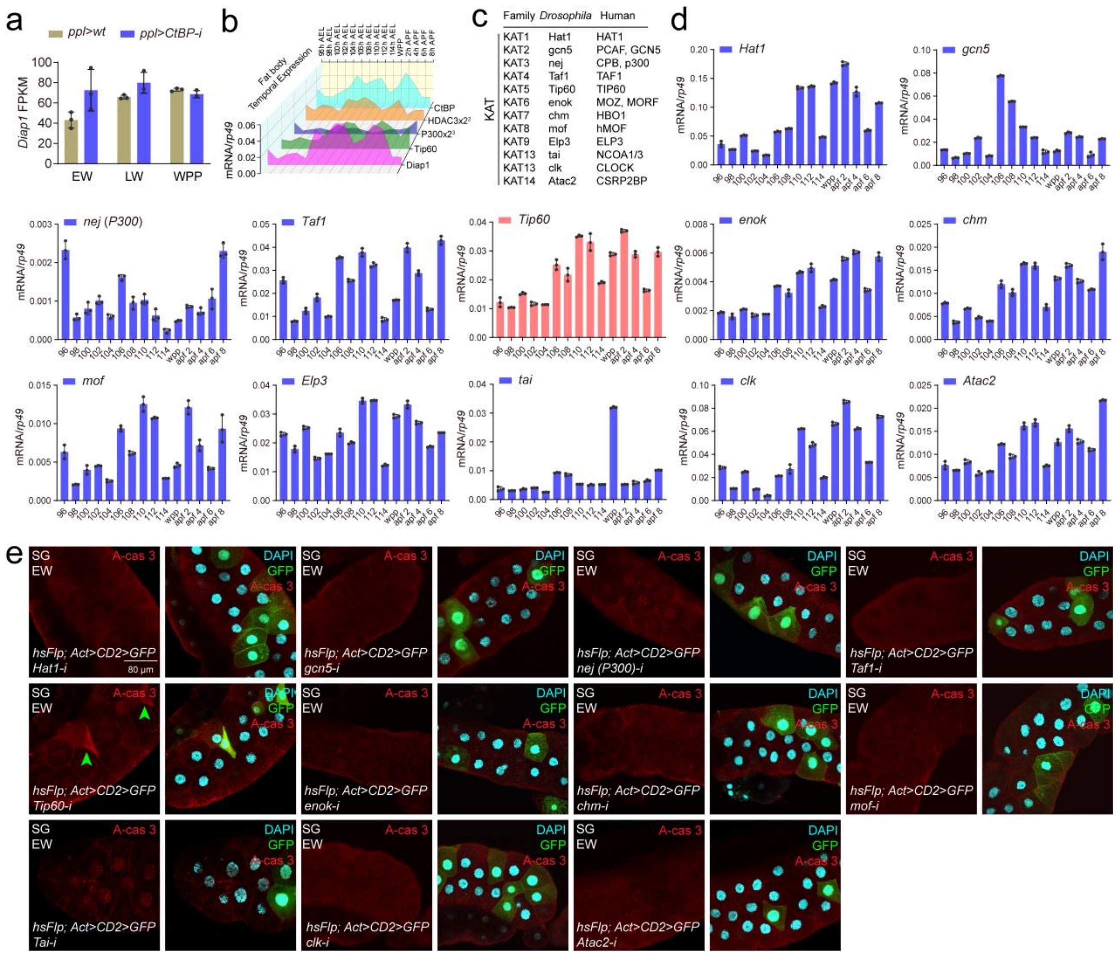

Supplementary Figure 9. RNAi screening to identify Tip60 regulating apoptosis, related to Figure 5

(**a**) FPKM values of *Diap1* after *CtBP-i* at three stages in the fat body using *ppl-Gal4*. The FPKM data was from RNA-seq in Fig. 1**f**. Mean ± SD; n = 3 independent samples.

(**b**) Relative transcriptional profiles (average of three repeated experiments) of *CtBP*, *HDAC3*, *Tip60*, *P300*, and *Diap1* in the fat body from 96 h AEL to 8 h APF. *Rp49* was used as the reference gene. To enhance the visual representation of gene expression dynamics, the Z-axis values for selected genes were amplified by a factor of 2^n.

(**c**) Homology of HATs in *Drosophila* and mammals.

(**d**) Developmental profiles of 12 *HATs* in the fat body from 96 h AEL to 8 h APF. Error bars indicate mean ± SEM. n = 3 independent samples.

(**e**) Identification of HATs that regulate apoptosis using Flp-out-mediated RNA-i in the salivary glands clone cells. GFP indicates clone cells with genes RNA-i. The green arrow represents cells undergoing apoptosis detected by the IF of A-cas 3.

Source data are provided as a Source Data file. The genotypes are provided in Supplementary Table 3.

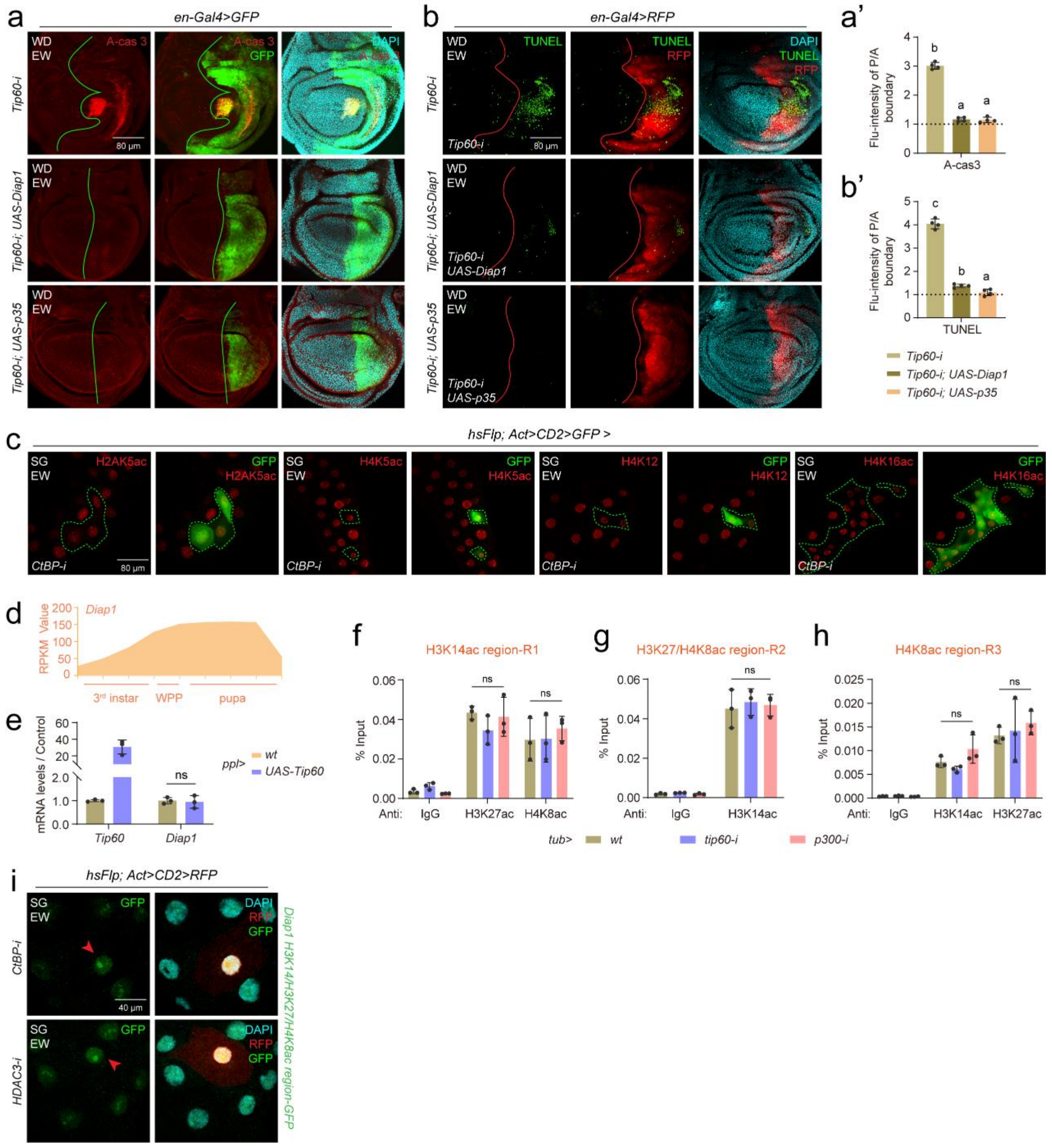

Supplementary Figure 10. Tip60/P300-CtBP/HDAC3 modulates H3K14ac/H3K27ac/H4K8ac homeodynamics in the *Diap1* promoters, related to Figure 5

(**a** and **a'**) After *Tip60-i*, *UAS-Tip60-i* & *UAS-Diap1*, and *UAS-Tip60-i* & *UAS-p35* at the EW, IF staining was performed to evaluate A-cas 3 in the wing disc (**a**). GFP region indicates the posterior (P) boundaries of the wing disc where gene RNA-i or overexpression is manipulated. The fluorescence intensity of A-cas 3 in the P boundary compared to the A boundary (**a'**). Mean ± SD; n = 4 independent wing disc. One-way ANOVA: different lowercase letters are significantly different ($P < 0.05$).

(**b** and **b'**) After *Tip60-i*, *UAS-Tip60-i* & *UAS-Diap1*, and *UAS-Tip60-i* & *UAS-p35* at the EW, TUNEL staining was performed to evaluate apoptosis in the wing disc (**b**). The fluorescence intensity of TUNEL in the P boundary compared to the A boundary (**b'**). Mean ± SD; n = 4 independent wing disc. One-way ANOVA: different lowercase letters are significantly different ($P < 0.05$).

(**c**) Evaluation of H2AK5ac, H4K5ac, H4K12ac, and H4K16ac after *CtBP-i* in the salivary glands using Flp-out line at the EW. GFP clone cells indicate *CtBP-i* cells.

(**d**) The orange area chart represents global development pattern of *Diap1* transcription. The RPKM data were obtained from FlyBase.

(**e**) Relative transcript level of *Diap1* after *UAS-Tip60* in the fat body at the EW using *ppl-Gal4*, *ppl-Gal4>wt* was used as control. Mean ± SD; n = 3 independent samples. Two-tailed paired *t* test: ns, not significant.

(**f-h**) Enrichment of H3K14ac/H3K27ac/H4K8ac in the R1 (**f**), R2 (**g**), and R3 (**h**) regions detected by ChIP-qPCR, after global *Tip60-i* and *P300-i* at 6 h APF using *tub-Gla4*, *tub-Gal4>wt* was used as control. Mean ± SD; n = 3 independent samples. Two-tailed paired *t* test: ns, not significant.

(**i**) Detection of the GFP signal indicating H3K14ac/H3K27ac/H4K8ac levels in the *Diap1* promoter in *CtBP-i* and *HDAC3-i* clone cells of the salivary glands at the EW using Flp-out. RFP clone cells indicate *CtBP-i* or *HDAC3-i* clone cells.

Source data are provided as a Source Data file. The genotypes are provided in Supplementary Table 3.

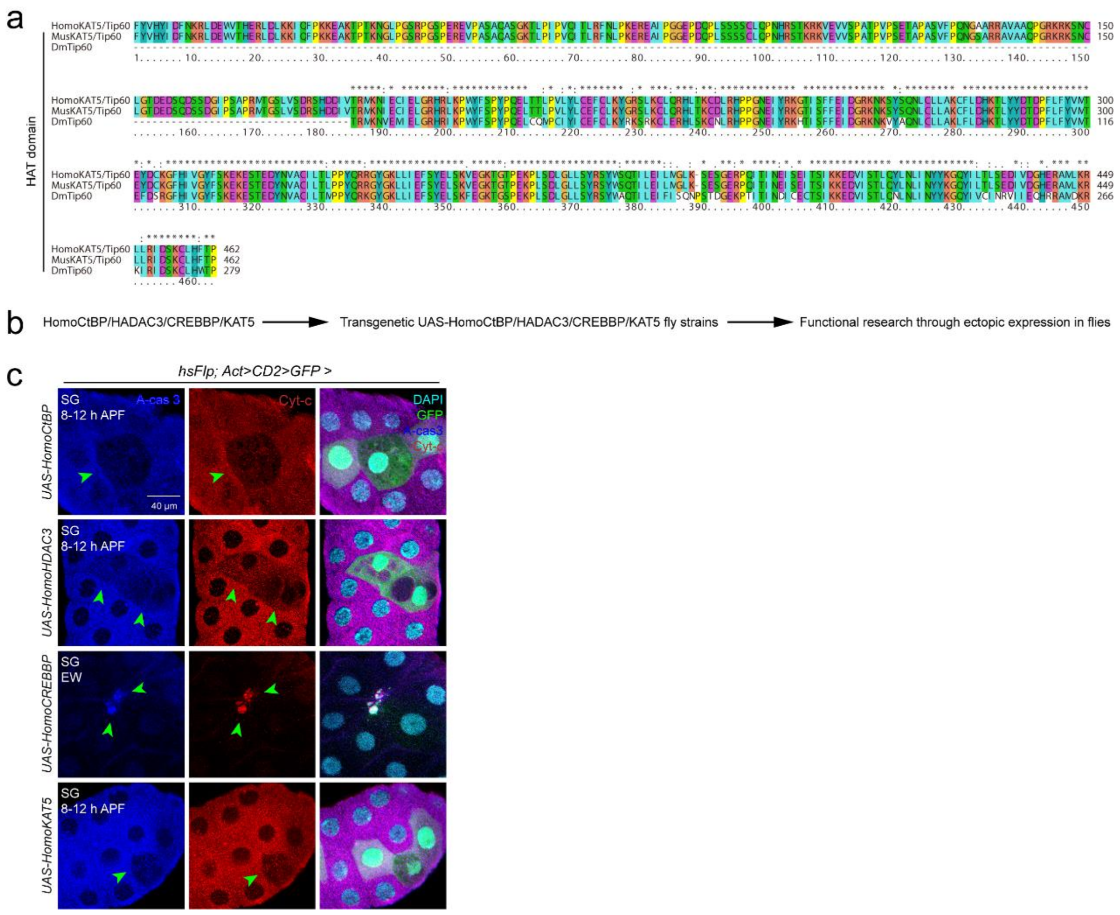

Supplementary Figure 11. Functional verification of human epigenetic factors in *Drosophila* tissues. related to Figure 6

(**a**) Homology analysis of the conserved HAT domains of KAT5 from *Drosophila* (Tip60), humans, and mice.

(**b**) Diagram showing *UAS-HomoCtBP*, *UAS-HomoHDAC3*, *UAS-HomoCREBBP*, and *UAS-HomoKAT5* transgenic flies.

(**c**) After the ectopic expression of *HomoCtBP*, *HomoHDAC3* at the EW, and the ectopic expression of *HomoCREBBP* and *HomoKAT5* at 8-12 h APF using Flp-out line, IF staining of Cyt-c and A-cas 3 in the salivary glands. GFP clone cells indicate genes with ectopic expression clone cells.

The genotypes are provided in Supplementary Table 3.

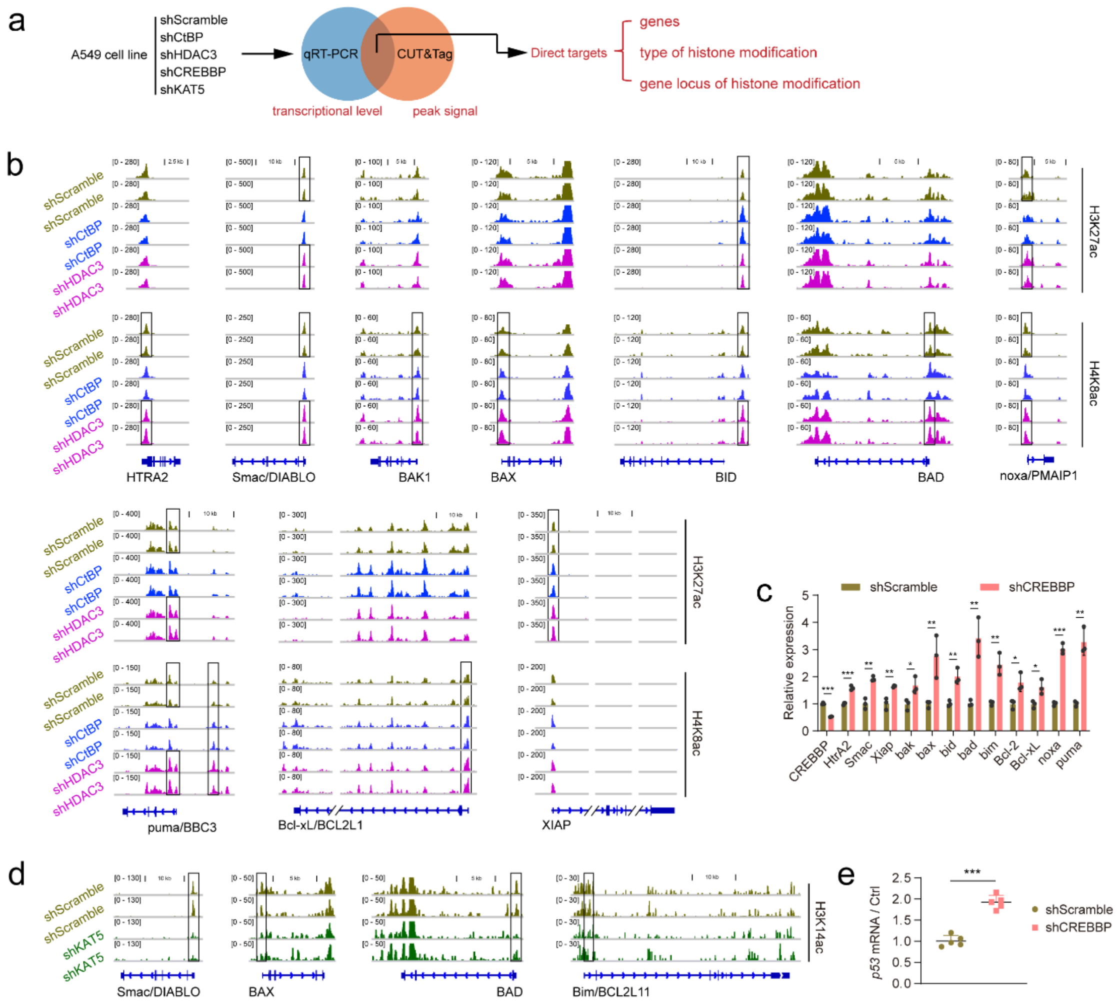

Supplementary Figure 12. *PAPs/MPAPs* and *IAPs/MIAPs* are directly regulated by histone acetylation homeodynamics in mammalian cells, related to Figure 6

(**a**) Strategy for identifying *PAPs/MPAPs* and *IAPs/MIAPs* directly regulated by histone acetylation modulated by HomoCtBP, HomoHDAC3, HomoCREBBP, and HomoKAT5.

(**b**) IGV tracks showing the up-regulated H3K27ac and H4K8ac levels in *PAPs/MPAPs* and *IAPs/MIAPs* promoters after knockdown of *HomoCtBP* and *HomoHDAC3*. The black rectangle represents peaks with elevated levels of H3K27ac or H4K8ac in the gene promoter region, and the peak signal values are in Fig. 6**d**.

(**c**) Evaluation of *PAPs/MPAPs* and *IAPs/MIAPs* transcription after knockdown of *HomoCREBBP* in A549 cells; *shScramble* was used as the control. Mean ± SD; n = 3 independent samples. Two-tailed paired *t* test: **p* <0.05, ***p* <0.01, ****p* <0.001.

(**d**) IGV tracks showing the down-regulated H3K14ac levels in *PAPs/MPAPs* and

IAPs/MIAPs promoters after knockdown of *HomoKAT5*. The black rectangle represents peaks with decreased levels of H3K14ac in the gene promoter region, and the peak signal values are in Fig. 6**e**.

(**e**) Detection of *p53* mRNA after knockdown *HomoCREBBP* in A549 cells; *shScramble* was used as the control. Mean ± SD; n = 5 independent samples. Two-tailed paired *t* test: ***p <0.001.

Source data are provided as a Source Data file.

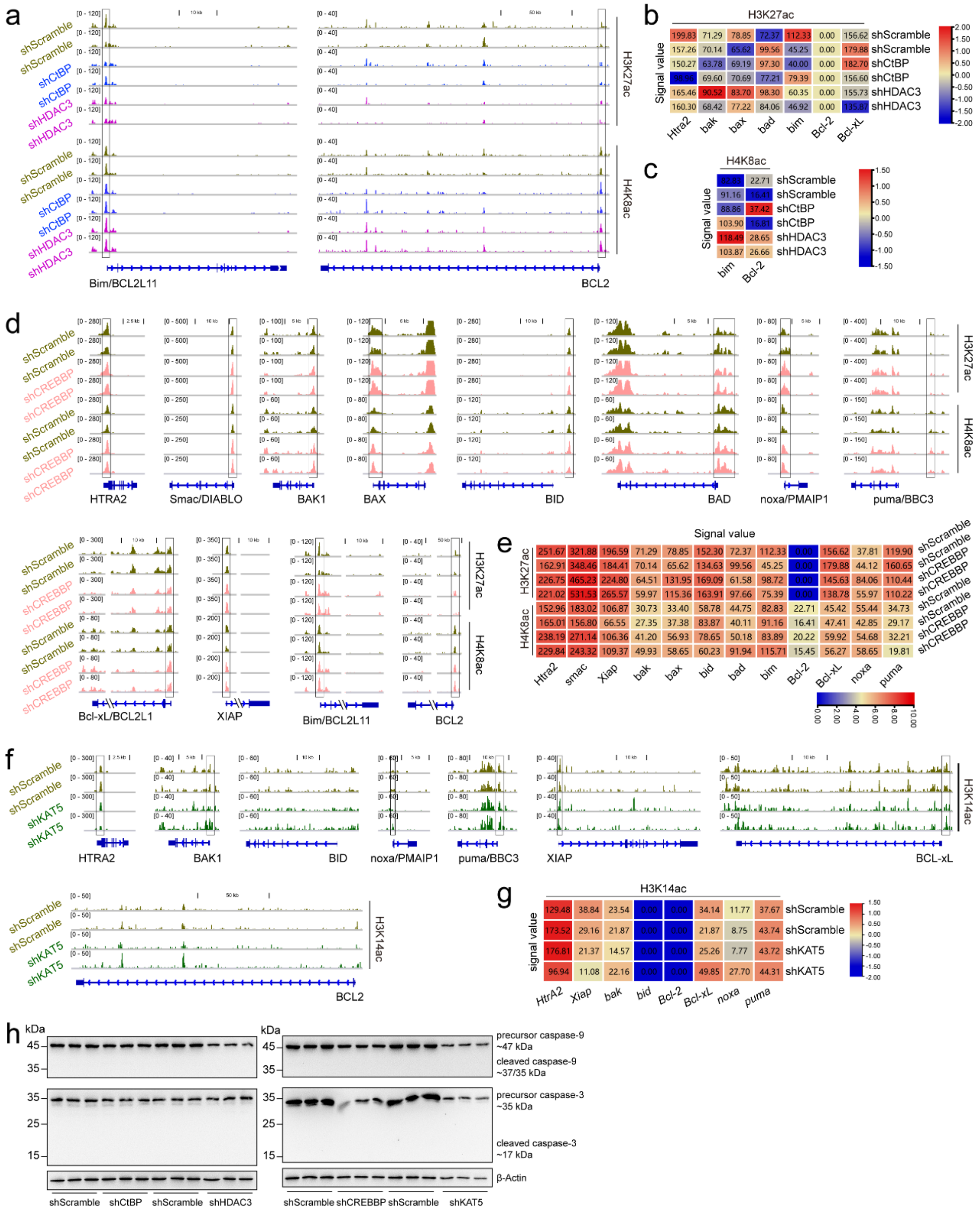

Supplementary Figure 13. *PAPs/MPAPs* and *IAPs/MIAPs* are not directly regulated by histone acetylation homeodynamics in mammalian cells, related to Figure 6

(**a**) IGV tracks showing unchanged H3K27ac and H4K8ac levels in *PAPs/MPAPs* and *IAPs/MIAPs* promoters after knockdown of *HomoCtBP* and *HomoHDAC3*. The black rectangle represents peaks with unchanged H3K27ac or H4K8ac levels in the gene promoter region.

(**b** and **c**) Heatmap showing the normalized unchanged peak signals (Col Scale) of H3K27ac (**b**) and H4K8ac (**c**) after knockdown of *HomoCtBP* and *HomoHDAC3*. Signal values of H3K27ac and H4K8ac in the squares are origin values enriched by CUT&Tag, and the enrichment regions were labeled with black rectangle in Fig. S13**a**.
(**d** and **e**) IGV tracks showing unchanged H3K27ac and H4K8ac levels (**d**) and heatmap of the normalized unchanged peak signals (**e**) in *PAPs/MPAPs* and *IAPs/MIAPs* promoters after knockdown of *HomoCREBBP*. Signal values of H3K27ac and H4K8ac in the squares are origin values enriched by CUT&Tag, and the enrichment regions were labeled with black rectangle.
(**f** and **g**) IGV tracks showing unchanged H3K14ac levels (**f**) and heatmap of the normalized unchanged peak signals (**g**) in *PAPs/MPAPs* and *IAPs/MIAPs* promoters after knockdown of *HomoKAT5*. Signal values of H3K14ac in the squares are origin values enriched by CUT&Tag, and the enrichment regions were labeled with black rectangle.
(**h**) Precursor caspase 9, cleaved caspase 9, precursor caspase 3, and cleaved caspase 3 detection after knockdown of *HomoCtBP*, *HomoHDAC3*, *HomoCREBBP*, and *HomoKAT5* in A549 cells; *shScramble* was used as the control.
Source data are provided as a Source Data file.

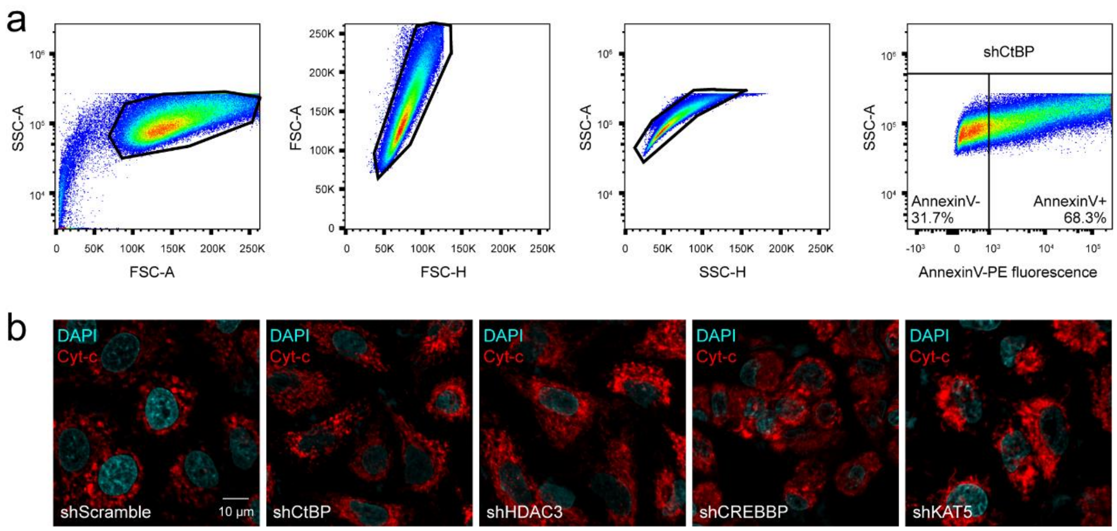

Supplementary Figure 14. Gating strategy of flow cytometry and detection of Cyt-c location, related to Figure 6

(**a**) Representative gating strategy to evaluate mitochondrial inner membrane potential, measured as Annexin V-phycoerythrin (PE) fluorescence by flow cytometry. The cell population was distinguished from the cell debris. After two rounds of single-cell group separation, the cells positive for Annexin V-PE were sorted. This gating strategy corresponds to control, *CtBP*, *HDAC3*, *CREBBP*, and *KAT5* knockdown cells in fig. 6**f**. (**b**) After knocking down *HomoCtBP*, *HomoHDAC3*, *HomoCREBBP*, and *HomoKAT5* in A549 cells, localization analysis of Cyt-c via IF staining.

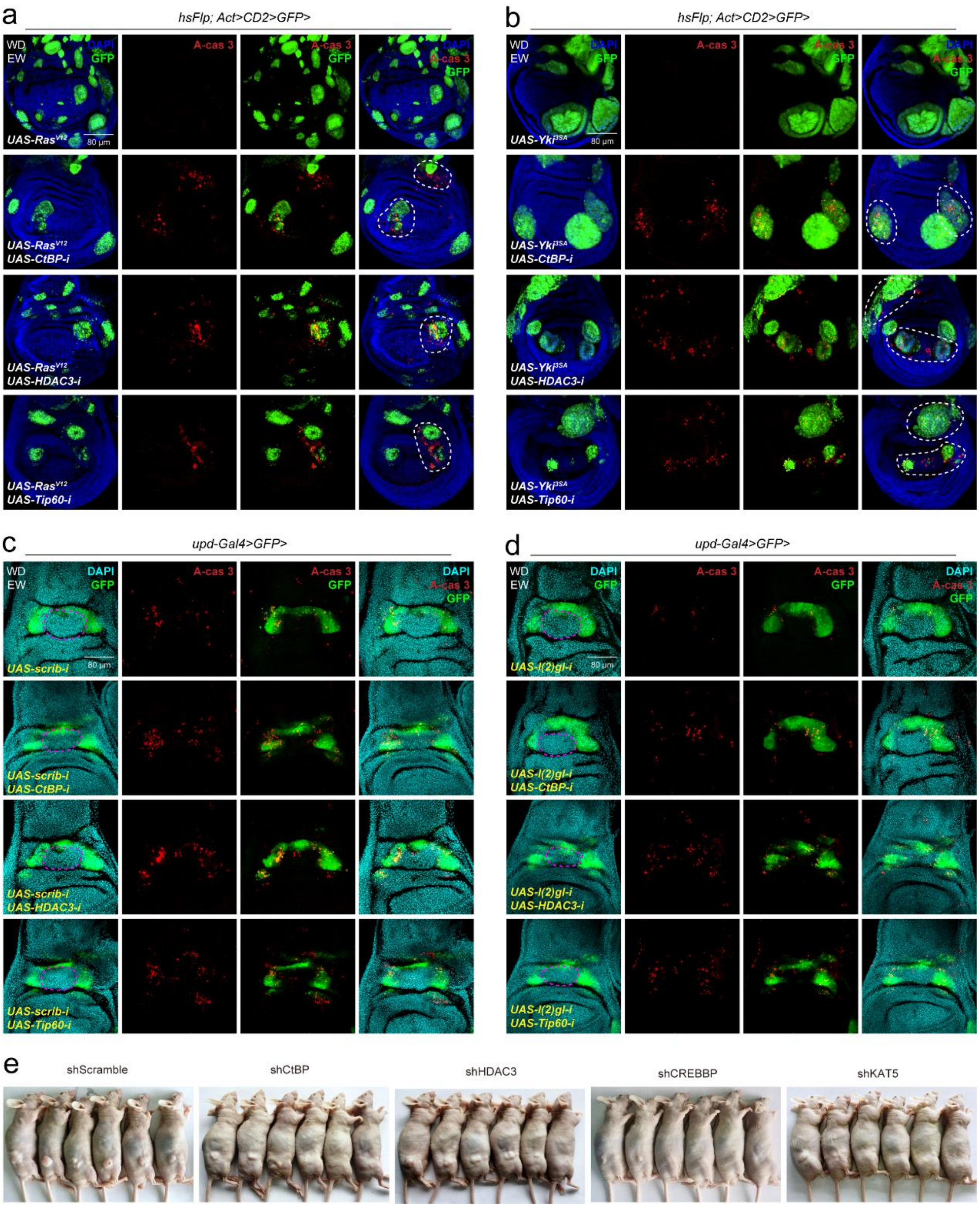

Supplementary Figure 15. Disruption of histone acetylation homeodynamics attenuates tumorigenesis in *Drosophila*, related to Figure 7

(**a** and **b**) Immunofluorescence staining of A-cas 3 after *CtBP-i*, *HDAC3-i*, and *Tip60-i* in *Ras*^{V12} (**a**) or *Yki*^{3SA} (**b**) overexpression-induced tumors in *Drosophila* wing discs using Flp-out line. Tumor cells were labelled by GFP.

(**c** and **d**) Immunofluorescence staining of A-cas 3 after *CtBP-i*, *HDAC3-i*, and *Tip60-i* in *scrib-i* (**c**) or *lgl2-i* (**d**)-induced tumors in *Drosophila* wing discs using *upd-Gal4*.

Tumors are marked with red dashed lines, which were between the two domains of the dorsal medial fold in the wing disc hinge region.

(**e**) After subcutaneous injection of stable *HomoCtBP*, *HomoHDAC3*, *HomoCREBBP*, and *HomoKAT5* knockdown A549 cells, *Scramble* knockdown A549 cells was used as the control group. The tumor-bearing mice in each group were photographed, n = 6 independent mice.

The genotypes are provided in Supplementary Table 3.

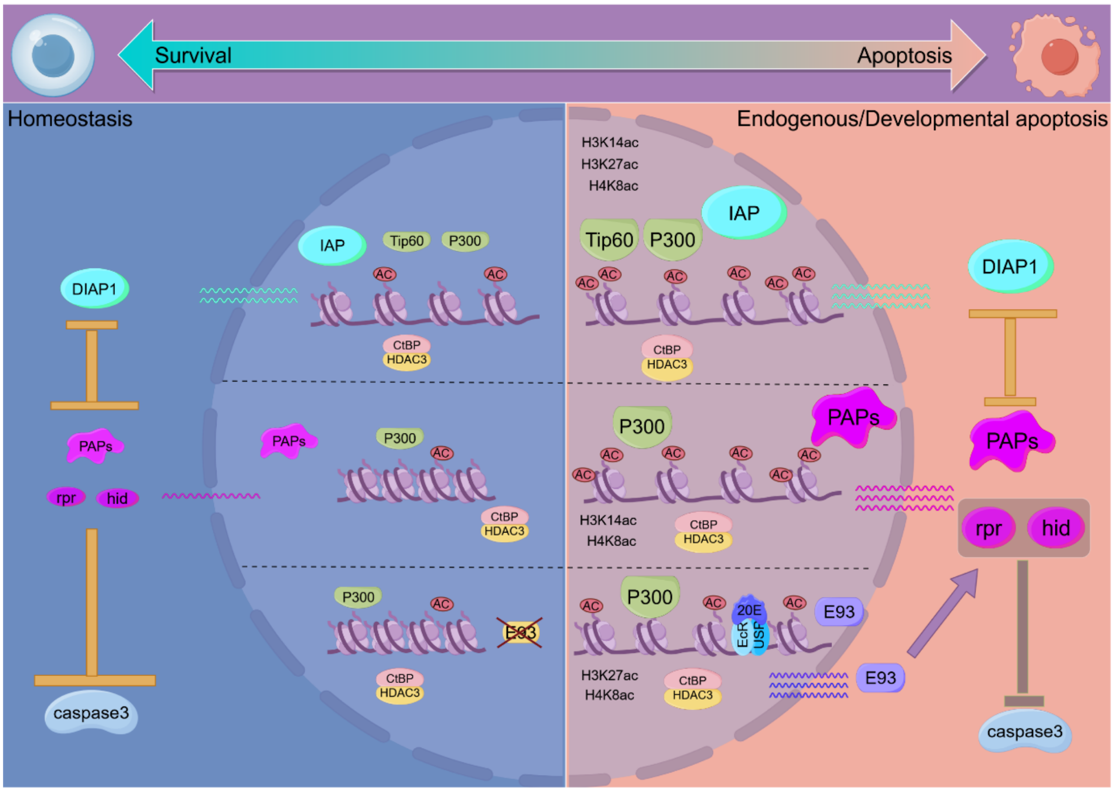

Supplementary Figure 16. P300/Tip60-CtBP/HDAC3 system navigates cell survival and apoptosis (By Figdraw)

In *Drosophia*, during tissue growth or in the absence of apoptosis, Tip60/P300-CtBP/HDAC3-mediated H3K14/H3K27/H4K8 acetylation occurs at the promoter of the *IAP* gene (*Diap1*), thereby sustaining a certain level of DIAP1 expression. Concurrently, P300-CtBP/HDAC3-mediated H3K14/H4K8 deacetylation at the promoter regions of *PAPs* (*rpr* and *hid*), and H3K27/H4K8 deacetylation at the enhancer/promoter of 20E-induced *E93* gene, keeping their expression at an undetectable or low level. Under these conditions, caspases remain inactive, effectively inhibiting apoptosis and preserving cellular homeostasis. In contrast, during tissue metamorphosis or when cell apoptosis is initiated, Tip60/P300-CtBP/HDAC3-mediated H3K14/H3K27/H4K8 acetylation maintains elevated levels of DIAP1 to prevent rapid cell death. Simultaneously, P300-CtBP/HDAC3-mediated acetylation promotes the robust expression of *PAPs* (*rpr* and *hid*) and *E93*. Consequently, caspases are activated, triggering endogenous or developmental apoptosis.

Supplementary Table 1. Information of Chemicals and Commercial Assays

Chemicals and Commercial Assays	SOURCE	IDENTIFIER
20-Hydroxyecdysone (20E)	Cayman	16145
MTA	MCE	HY-16938
PU139	MCE	HY-124696
C646	MCE	HY-13823
4',6-diamidino-2-phenylindole (DAPI)	Beyotime	C1002
Schneider's Drosophila medium	Sigma	21720
Dulbecco’s Modified Eagle Medium	Gibco	11995500BT
GlutaMAX	Gibco	35050079
penicillin-streptomycin solution	Gibco	15070063
heat-inactivated fetal bovine serum	Gibco	10099
protease inhibitor cocktail	Thermo	78429
TRIzol Reagent	Invitrogen	15596026
M-MLV reverse transcriptase	Takara	2641Q
RNase inhibitor	Takara	2313Q
Hieff qPCR SYBR Green Master Mix	Yeasen	11201ES03
NP-40 lysis buffer	Beyotime	P0013F
Caspase 3 Activity Assay Kit	Beyotime	C1115
One Step TUNEL Apoptosis Assay Kit	Beyotime	C1086
Plasmid Midi Kit	QIAGEN	12145
Effectene Transfection Reagent	QIAGEN	301427
Dual-Luciferase Reporter Assay System	Promega	E1960
Protein A/G Agarose beads	Thermo	26161
NovoNGS CUT&Tag 3.0 High-Sensitivity Kit	Novoprotein	N259-YH01
Pierce agarose ChIP kit	Thermo	26156
Cell Mitochondria Isolation Kit	Beyotime	C3601
Annexin V-PE Apoptosis Detection Kit	Beyotime	C1065M
Mitochondrial Membrane Potential Assay Kit with TMRE	Beyotime	C2001S

Supplementary Table 2. Information of Fly Stains

Fly stains	SOURCE	IDENTIFIER
w^{1118}	Bloomington Stock Center	BDSC_3605
tub-Gal4	Tsinghua University Fly Center	TB00129
ppl-Gal4	Bloomington Stock Center	BDSC_58768
en-Gal4/CyO	Kyoto Stock Center	KSC108024
tub-GAL80^{ts}*; TM2/TM6B*	Bloomington Stock Center	BDSC_7019
tub-GAL80^{ts}*/FM7c*	Bloomington Stock Center	BDSC_7016
ptc-Gal4	Tsinghua University Fly Center	THJ0201
hsFlp; Act5c>CD2>Gal4>UAS-GFP	A gift from Dr. Zizhang Zhou	N/A
hsFlp; Act5c>CD2>Gal4>UAS-RFP	A gift from Dr. Zizhang Zhou	N/A
FRT82B	Bloomington Stock Center	BDSC_1459
FRT82B, tubP-Gal80/TM6B	Bloomington Stock Center	BDSC_5135
UAS-dicer-2	Bloomington Stock Center	BDSC_24650
UAS-CtBP RNAi	Vienna Drosophila Resource Center	v107313
UAS-CtBP RNAi	Vienna Drosophila Resource Center	v37609
UAS-CtBP-V5	This study	N/A
UAS-E93 RNAi	Bloomington Stock Center	BDSC_57868
UAS-E93 RNAi	Vienna Drosophila Resource Center	V104390
UAS-E93	FlyORF	F000587
UAS-Diap1	FlyORF	F001601
UAS-HDAC3 RNAi	Vienna Drosophila Resource Center	V20814
UAS-HDAC3 RNAi	Bloomington Stock Center	BDSC_31633
UAS-HDAC3-Flag	This study	N/A
UAS-P300 RNAi	Vienna Drosophila Resource Center	v102885
UAS-P300	Bloomington Stock Center	BDSC_32573
UAS-EcR-B1	Bloomington Stock Center	BDSC_6469
UAS-EcR-B1^{DN}	Bloomington Stock Center	BDSC_6869
E93^{-}*/TM6B (E93 mutant)*	This lab	N/A
upd-Gal4; Sco/CyO	Tsinghua University Fly Center	THJ0196
upd-Gal4; UAS-GFP/CyO	Tsinghua University Fly Center	THJ0197
FRT82B, tubP-Gal80/TM6B	Bloomington Stock Center	BDSC_5135
UAS-Tip60 RNAi	Tsinghua University Fly Center	THU3470
UAS-Tip60 RNAi	Bloomington Stock Center	BDSC_35243
UAS-Tip60	FlyORF	F000567
UAS-p35/CyO; Sb/TM6B	Tsinghua University Fly Center	THJ0122
UAS-Ras^{V12}*; Sb/TM6B*	Tsinghua University Fly Center	THJ0150
UAS-Yki^{3SA}	A gift from Dr. Zizhang Zhou	N/A
UAS-Scrib RNAi	Vienna Drosophila Resource Center	v45555
UAS-l(2)gl	Vienna Drosophila Resource Center	v109604
UAS-Rpd3 RNAi	Tsinghua University Fly Center	THU1953
UAS-Rpd3 RNAi	Tsinghua University Fly Center	THU0695
UAS-HDAC4 RNAi	Tsinghua University Fly Center	THU0620

UAS-HDAC6 RNAi	Tsinghua University Fly Center	THU1981
UAS-HDAC6 RNAi	Tsinghua University Fly Center	TH201500467.S
UAS-HDACX RNAi	Tsinghua University Fly Center	THU0926
UAS-Sir2 RNAi	Tsinghua University Fly Center	THU1967
UAS-Sir2 RNAi	Tsinghua University Fly Center	THU0927
UAS-Sirt2 RNAi	Tsinghua University Fly Center	THU1905
UAS-Sirt2 RNAi	Tsinghua University Fly Center	THU0928
UAS-Sirt4 RNAi	Tsinghua University Fly Center	THU1327
UAS-Sirt4 RNAi	Tsinghua University Fly Center	THU1969
UAS-Sirt6 RNAi	Tsinghua University Fly Center	THU1392
UAS-Sirt6 RNAi	Tsinghua University Fly Center	THU1992
UAS-Sirt7 RNAi	Tsinghua University Fly Center	THU0929
UAS-Sirt7 RNAi	Tsinghua University Fly Center	THU1978
UAS-Hat1 RNAi	Tsinghua University Fly Center	THU3701
UAS-gcn5 RNAi	Tsinghua University Fly Center	THU1324
UAS-gcn5 RNAi	Tsinghua University Fly Center	THU3605
UAS-Taf1 RNAi	Tsinghua University Fly Center	THU0867
UAS-Taf1 RNAi	Tsinghua University Fly Center	TH201500889.S
UAS-enok RNAi	Tsinghua University Fly Center	THU5462
UAS-chm RNAi	Tsinghua University Fly Center	THU0930
UAS-chm RNAi	Tsinghua University Fly Center	THU2412
UAS-mof RNAi	Tsinghua University Fly Center	THU1005
UAS-mof RNAi	Tsinghua University Fly Center	THU5818
UAS-tai RNAi	Vienna Drosophila Resource Center	V330416
UAS-tai RNAi	Tsinghua University Fly Center	THU5870
UAS-clk RNAi	Tsinghua University Fly Center	THU4958
UAS-clk RNAi	Tsinghua University Fly Center	THU5790
UAS-Atac2 RNAi	Tsinghua University Fly Center	THU1079
E93 H3K27ac/H4K8ac enhancer-GFP	This study	N/A
rpr H3K14/H4K8ac promoter-GFP	This study	N/A
hid H3K14/H4K8ac promoter-GFP	This study	N/A
grim promoter-GFP	This study	N/A
Diap1 H3K14ac/H3K27ac/H4K8ac promoter-GFP	This study	N/A
UAS-DmCtBP-V5	This study	N/A
UAS-DmHDAC3-Flag	This study	N/A
UAS-HomoCtBP	This study	N/A
UAS-HomoHDAC3	This study	N/A
UAS-HomoCREBBP	This study	N/A
UAS-HomoKAT5	This study	N/A

Supplementary Table 3. Genotypes and Genetic Manipulation of *Drosophila*

Figure 1		
**a**	*+/+; ppl-Gal4/+; +/+*	25℃
	+/+; ppl-Gal4/+; UAS-CtBP RNAi/+	
**b-e**	*+/+; ppl-Gal4/+; +/+*	29℃
	+/+; ppl-Gal4/+; UAS-CtBP RNAi/+	
**f**	*+/+; ppl-Gal4/+; +/+*	25℃
	+/+; ppl-Gal4/+; UAS-CtBP RNAi/+	
**h**	*+/+; ppl-Gal4/+; +/+*	29℃
	+/+; ppl-Gal4/UAS-CtBP RNAi; +/+	
	+/+; ppl-Gal4/+; UAS-E93/+	
	+/+; ppl-Gal4/UAS-CtBP RNAi; UAS-E93 RNAi/+	
	+/+; ppl-Gal4/UAS-CtBP RNAi; UAS-Diap1/+	
**i**	*hsFlp/+; UAS-dicer/+; Act5c>CD2>Gal4>UAS-GFP*	
	hsFlp/+; UAS-CtBP RNAi/UAS-dicer; Act5c>CD2>Gal4>UAS-GFP/+	
	hsFlp/+; UAS-dicer/+; Act5c>CD2>Gal4>UAS-GFP/UAS-E93	
	hsFlp/+; UAS-CtBP RNAi/UAS-dicer; Act5c>CD2>Gal4>UAS-GFP/UAS-E93 RNAi	
	hsFlp/+; UAS-CtBP RNAi/UAS-dicer; Act5c>CD2>Gal4>UAS-GFP/UAS-Diap1	
**k**	*hsFlp/+; UAS-CtBP RNAi/UAS-dicer; Act5c>CD2>Gal4>UAS-GFP/+*	
	hsFlp/+; UAS-dicer/+; Act5c>CD2>Gal4>UAS-GFP/UAS-E93	
	hsFlp/+; UAS-CtBP RNAi/UAS-dicer; Act5c>CD2>Gal4>UAS-GFP/UAS-E93 RNAi	
	hsFlp/+; UAS-CtBP RNAi/UAS-dicer; Act5c>CD2>Gal4>UAS-GFP/UAS-Diap1	
**m**	*+/+; en-Gal4>UAS-GFP/UAS-CtBP RNAi; +/+*	
	+/+; Gal80ts, en-Gal4>UAS-GFP/+; UAS-E93/+	18-

		29°C
	+/+; en-Gal4>UAS-GFP/UAS-CtBP RNAi; UAS-E93 RNAi/+	29°C
	+/+; en-Gal4>UAS-GFP/UAS-CtBP RNAi; UAS-Diap1/+	

In **a**, the statistics of pupation time and rate was from 120 h after egg laying (AEL).

In **d** and **e**, the fat body of were dissected at early wandering (EW) stage.

In **f**, the fat body were dissected at EW, late wandering (LW), and white pre-pupal (WPP) stage, respectively.

In **i** and **k**, the progeny were heat shocked at 37°C for 15 min at 24 h AEL and were then cultured at 29°C to EW stage before dissection.

In **h**, the progeny were placed at 29°C from egg laying to EW stage before dissection.

In **m**, except the progeny of *+/+; Gal80ts, en-Gal4>UAS-GFP/+; UAS-E93/+* were cultured at 18°C from egg laying until the mid 3rd instar, and then were cultured at 29°C to EW stage before dissection, other progeny were placed at 29°C from egg laying to EW stage before dissection.

**Figure S1, related to Figure 1**		
**c**, **i-k**	*w^{1118}*	25°C
**d-h**	*+/+; tub-Gal4/+; +/+*	
	+/+; tub-Gal4/+; UAS-CtBP RNAi/+	

In C, the fat body were dissected from 96 h AEL to 8 h APF at intervals of 2 hours.

In D, the statistics of pupation time and rate was from 120 h AEL.

In H, the fat body were dissected at EW stage.

In I-K, the fat body were dissected from 96 h AEL to 8 h APF; the salivary glands were dissected from 96 h AEL to 8 h APF; the wing disc were dissected from 96 h AEL to 6 h APF.

**Figure S2, related to Figure 1**		
**a-d**	*+/+; ppl-Gal4/+; +/+*	25°C
	+/+; ppl-Gal4/+; UAS-CtBP RNAi/+	
**e**	*hsFlp/+; UAS-dicer/+; Act5c>CD2>Gal4>UAS-GFP*	29°C
**f**	*+/+; en-Gal4>UAS-GFP/+; +/+*	29°C
**g**, **g'**	See Fig. 1**m** and Fig. S2**f**	

In **a-d**, the fat body were dissected at EW, LW, and WPP stages, respectively.

In **e**, see Fig.1**k**. In **f**, see Fig.1**m**. In **g**, see Fig.1**m**.

Figure 2		
**a**	*+/+; ppl-Gal4; +/+*	29°C
	+/+; ppl-Gal4/UAS-HDAC3 RNAi; +/+	
	+/+; ppl-Gal4/UAS-HDAC3 RNAi; UAS-E93 RNAi/+	
	+/+; ppl-Gal4/UAS-HDAC3 RNAi; UAS-Diap1/+	
**b**	*hsFlp/+; UAS-dicer/+; Act5c>CD2>Gal4>UAS-GFP/+*	
	hsFlp/+; UAS-HDAC3 RNAi/UAS-dicer; Act5c>CD2>Gal4>UAS-GFP/+	
	hsFlp/+; UAS-HDAC3 RNAi/UAS-dicer; Act5c>CD2>Gal4>UAS-GFP/UAS-E93 RNAi	
	hsFlp/+; UAS-HDAC3 RNAi/UAS-dicer; Act5c>CD2>Gal4>UAS-GFP/UAS-Diap1	
**c**	*+/+; ppl-Gal4/+; +/+*	25°C
	+/+; ppl-Gal4/UAS-HDAC3 RNAi; +/+	
**d**	*hsFlp/+; UAS-HDAC3 RNAi/UAS-dicer; Act5c>CD2>Gal4>UAS-GFP/+*	29°C
	hsFlp/+; UAS-HDAC3 RNAi/UAS-dicer; Act5c>CD2>Gal4>UAS-GFP/UAS-E93 RNAi	
	hsFlp/+; UAS-HDAC3 RNAi/UAS-dicer; Act5c>CD2>Gal4>UAS-GFP/UAS-Diap1	
**e**	*hsFlp/+; UAS-CtBP-V5/+; Act5c>CD2>Gal4>UAS-GFP/+*	25°C
	hsFlp/+; +/+; Act5c>CD2>Gal4>UAS-GFP/UAS-HDAC3-Flag	
	hsFlp/+; UAS-CtBP-V5/+; Act5c>CD2>Gal4>UAS-GFP/UAS-HDAC3-Flag	

In **b**, **d**, and **e**, the progeny were heat shocked at 37°C for 15 min at 24 h AEL and then were cultured at the indicated temperature to the indicated stages before dissection.
In **a**, **b**, and **d**, tissues were dissected at EW stage.
In **e**, tissues were dissected at 8-12 h APF.
In **c**, the fat body were dissected at EW stage.

Figure S3, related to Figure 2

**c**, **d**	*w^{1118}*	25°C
**e**	*hsFlp/+; UAS-dicer/+; Act5c>CD2>Gal4>UAS-GFP/UAS-Rpd3 RNAi*	29°C
	hsFlp/+; UAS-dicer/+; Act5c>CD2>Gal4>UAS-GFP/UAS-HDAC3 RNAi	
	hsFlp/+; UAS-dicer/+; Act5c>CD2>Gal4>UAS-GFP/UAS-HDAC4 RNAi	
	hsFlp/+; UAS-dicer/+; Act5c>CD2>Gal4>UAS-GFP/UAS-HDAC6 RNAi	
	hsFlp/+; UAS-dicer/+; Act5c>CD2>Gal4>UAS-GFP/UAS-HDACX RNAi	
	hsFlp/+; UAS-dicer/+; Act5c>CD2>Gal4>UAS-GFP/UAS-Sir2 RNAi	
	hsFlp/+; UAS-dicer/+; Act5c>CD2>Gal4>UAS-GFP/UAS-Sirt2 RNAi	
	hsFlp/+; UAS-dicer/+; Act5c>CD2>Gal4>UAS-GFP/UAS-Sirt4 RNAi	
	hsFlp/+; UAS-dicer/+; Act5c>CD2>Gal4>UAS-GFP/UAS-Sirt6 RNAi	
	hsFlp/+; UAS-dicer/+; Act5c>CD2>Gal4>UAS-GFP/UAS-Sirt7 RNAi	
In **c** and **d**, the fat body were dissected from 96 h AEL to 8 h APF at intervals of 2 hours.<br>In **e**, the progeny were heat shocked at 37°C for 15 min at 24 h AEL and then were cultured at 29°C to EW stage before dissection.		

**Figure S4, related to Figure 2**		
**a**, **b**	*+/+; en-Gal4>UAS-GFP/UAS-HDAC3 RNAi; +/+*	29°C
	+/+; en-Gal4>UAS-GFP/UAS-HDAC3 RNAi; UAS-E93 RNAi/+	
	+/+; en-Gal4>UAS-GFP/UAS-HDAC3 RNAi; UAS-Diap1/+	
**c**, **d**	*w^{1118}*	25°C
**e**	*hsFlp/+; UAS-CtBP-V5/+; Act5c>CD2>Gal4>UAS-GFP/UAS-HDAC3-Flag*	
In **a** and **b**, tissues were dissected at EW stage.<br>In **c** and **d**, the salivary gland were dissected at 96 h AEL, W, WPP, 6 h APF, 12 h APF, and 14 h APF stages.<br>In **e**, the progeny were heat shocked at 37°C for 15 min at 24 h AEL and were then cultured at 25°C to EW stage before dissection.		

**Figure 3**		
**a**	*hsFlp/+; UAS-P300 RNAi/UAS-dicer; Act5c>CD2>Gal4>UAS-GFP/+*	29°C

	hsFlp/+; UAS-P300 RNAi/UAS-dicer; Act5c>CD2>Gal4>UAS-GFP/UAS-E93	
**b**	*+/+; ppl-Gal4/+; +/+*	
	+/+; ppl-Gal4/+; UAS-P300/+	
	+/+; ppl-Gal4/+; UAS-P300::UAS-E93 RNAi/+	
**c**, **d**	*hsFlp/+; UAS-dicer/+; Act5c>CD2>Gal4>UAS-GFP/UAS-P300*	
	hsFlp/+; UAS-dicer/+; Act5c>CD2>Gal4>UAS-GFP/UAS-P300::UAS-E93 RNAi	
	hsFlp/+; UAS-dicer/+; Act5c>CD2>Gal4>UAS-GFP/UAS-P300::UAS-Diap1	
**f**	w^{1118}	25℃
**h**	*+/+; tub-Gal4/+; +/+*	
	+/+; tub-Gal4/UAS-P300 RNAi; +/+	
**i**	*hsFlp/+; E93 H3K27ac/H4K8ac enhancer-GFP/UAS-dicer; Act5c>CD2>Gal4>UAS-RFP/UAS-CtBP RNAi*	
	hsFlp/+; E93 H3K27ac/H4K8ac enhancer-GFP/UAS-dicer; Act5c>CD2>Gal4>UAS-RFP/UAS-HDAC3 RNAi	
	hsFlp/+; E93 H3K27ac/H4K8ac enhancer-GFP/UAS-dicer; Act5c>CD2>Gal4>UAS-RFP/UAS-P300	
	hsFlp/+; E93 H3K27ac/H4K8ac enhancer-GFP/UAS-dicer; Act5c>CD2>Gal4>UAS-RFP/UAS-EcR-B1	
**j**	*hsFlp/+; E93 H3K27ac/H4K8ac enhancer-GFP/UAS-CtBP; Act5c>CD2>Gal4>UAS-RFP/+*	25℃
	hsFlp/+; E93 H3K27ac/H4K8ac enhancer-GFP/+; Act5c>CD2>Gal4>UAS-RFP/UAS-HDAC3	
	hsFlp/+; E93 H3K27ac/H4K8ac enhancer-GFP/UAS-P300 RNAi; Act5c>CD2>Gal4>UAS-RFP/+	
	hsFlp/+; E93 H3K27ac/H4K8ac enhancer-GFP/UAS-EcR-$B1^{DN}$*; Act5c>CD2>Gal4>UAS-RFP/+*	

In **a**, **c**, **d**, **i**, and **j**, the progeny were heat shocked at 37°C for 15 min at 24 h AEL and were then cultured at the indicated temperature to the indicated stages before dissection. In **a** and **j**, 8-12 h APF; In **b**, **c**, **d**, and **i**, EW stage.<br>In **f**, the whole body of progeny were collected at 96 h AEL and 6 h APF; In **h**, the whole body of progeny were collected at 6 h APF.

**Figure S5, related to Figure 3**		
**c**	*+/+; ppl-Gal4/+; +/+*	29°C
	+/+; ppl-Gal4/UAS-P300 RNAi; +/+	
**d**, **e**	*+/+; Gal80ts, ppl-Gal4/+; +/+*	18-29°C
	+/+; Gal80ts; ppl-Gal4/+; UAS-P300/+	
**f**	*hsFlp/+; UAS-dicer/+; Act5c>CD2>Gal4>UAS-GFP*	29°C
**g**, **h**	*+/+; Gal80ts, en-Gal4>UAS-GFP/+; +/+*	18-29°C
	+/+; Gal80ts, en-Gal4>UAS-GFP/+; UAS-P300/+	
	+/+; Gal80ts, en-Gal4>UAS-GFP/+; UAS-P300::UAS-E93 RNAi/+	
	+/+; Gal80ts, en-Gal4>UAS-GFP/+; UAS-P300::UAS-Diap1/+	
In **c**, the progeny were cultured at 29°C to EW stage before dissection.<br>In **d** and **e**, the progeny were cultured at 18°C from egg laying until the mid 3rd instar, and then were cultured at 29°C to EW stage before dissection.<br>In **f**, the progeny were heat shocked at 37°C for 15 min at 24 h AEL and were then cultured at 29°C to 8-12 h APF before dissection.<br>In **g** and **h**, the progeny were cultured at 18°C from egg laying until the mid 3rd instar, and then were cultured at 29°C to EW stage before dissection.		

**Figure S6, related to Figure 3**		
**a**, **c**	*hsFlp/+; UAS-dicer/UAS-HDAC3 RNAi; Act5c>CD2>Gal4>UAS-GFP/+*	29°C
**b**	*hsFlp/+; UAS-dicer/+; Act5c>CD2>Gal4>UAS-GFP/UAS-CtBP RNAi*	
**e**	w^{1118}	25°C
**f-h**	*+/+; tub-Gal4/+; +/+*	
	+/+; tub-Gal4/UAS-P300 RNAi; +/+	
**i**	*+/+; tub-Gal4/+; +/+*	

	+/+; tub-Gal4/+; +/UAS-CtBP RNAi	
In **a-c**, the progeny were heat shocked at 37°C for 15 min at 24 h AEL and were then cultured at 29°C to EW stage before dissection.<br>In **e**, the whole body of progeny were collected at 96 h AEL and 6 h APF.<br>In **f-h**, the whole body of progeny were collected at 6 h APF.<br>In **i**, the whole body of progeny were collected at the EW.		

**Figure 4**		
**a**	*+/+; ppl-Gal4/+; E93^{-}/E93^{-}*	29°C
	+/+; ppl-Gal4/UAS-CtBP RNAi; E93^{-}/E93^{-}	
	+/+; ppl-Gal4/UAS-CtBP RNAi; UAS-Diap1::E93^{-}/E93^{-}	
	+/+; ppl-Gal4/UAS-HDAC3 RNAi; E93^{-}/E93^{-}	
	+/+; ppl-Gal4/UAS-HDAC3 RNAi; UAS-Diap1::E93^{-}/E93^{-}	
**b**	*+/+; Gal80ts, ppl-Gal4/+; E93^{-}/E93^{-}*	18-29°C
	+/+; Gal80ts, ppl-Gal4/+; UAS-P300::E93^{-}/E93^{-}	
	+/+; Gal80ts, ppl-Gal4; UAS-P300::UAS-Diap1::E93^{-}/E93^{-}	
**d**	*hsFlp/+; ptc>GFP/UAS-CtBP RNAi; FRT82B, tubP-Gal80/FRT82B, E93^{-}*	29°C
	hsFlp/+; ptc>GFP/UAS-CtBP RNAi; FRT82B, tubP-Gal80/UAS-Diap1::FRT82B, E93^{-}	
	hsFlp/+; ptc>GFP/UAS-HDAC3 RNAi; FRT82B, tubP-Gal80/FRT82B, E93^{-}	
	hsFlp/+; ptc>GFP/UAS-HDAC3 RNAi; FRT82B, tubP-Gal80/UAS-Diap1::FRT82B, E93^{-}	
	hsFlp/+; ptc>GFP/+; FRT82B, tubP-Gal80/UAS-P300::FRT82B, E93^{-}	25°C
	hsFlp/+; ptc>GFP/+; FRT82B, tubP-Gal80/UAS-Diap1::UAS-P300::FRT82B, E93^{-}	
**e**	*+/+; ppl-Gal4/+; E93^{-}/E93^{-}*	29°C
	+/+; ppl-Gal4/UAS-CtBP RNAi; E93^{-}/E93^{-}	
	+/+; ppl-Gal4/UAS-HDAC3 RNAi; E93^{-}/E93^{-}	
**f**	*+/+; Gal80ts, ppl-Gal4/+; E93^{-}/E93^{-}*	18-

	+/+; Gal80ts, ppl-Gal4/+; UAS-P300::E93^{-}*/E93*$^{-}$	29°C
**g**, **h**	*w*^{1118}	25°C
**i**, **j**	*+/+; tub-Gal4/+; E93*$^{-}$*/E93*$^{-}$	
	+/+; tub-Gal4/UAS-CtBP RNAi; E93^{-}*/E93*$^{-}$	
	+/+; tub-Gal4/UAS-HDAC3 RNAi; E93^{-}*/E93*$^{-}$	
	+/+; tub-Gal4/UAS-P300 RNAi; E93^{-}*/E93*$^{-}$	

In **a**, **b**, and **e**, the fat body of progeny were dissected at EW stage. In **f**, the progeny were cultured at 18°C from egg laying until the mid 3rd instar, and then were cultured at 29°C to EW stage before fat body dissection.

In **d**, the progeny were heat shocked at 37°C for 60 min at 8-10 h AEL and were then cultured at the indicated temperature to EW stage before dissection.

In **g** and **h**, the whole body of progeny were collected at 96 h AEL and 6 h APF.

In **i** and **j**, the progeny of *CtBP RNAi* and *HDAC3 RNAi* were collected at EW stage; the progeny of *P300 RNAi* were collected at 6 h APF.

Figure S7, related to Figure 4

**b**	*w*^{1118}	25°C
**c**	*+/+; +/+; E93*$^{-}$*/E93*$^{-}$	
**d**, **g**	*+/+; en-Gal4>UAS-GFP/UAS-CtBP RNAi; E93*$^{-}$*/E93*$^{-}$	29°C
	+/+; en-Gal4>UAS-GFP/UAS-CtBP RNAi; UAS-Diap1::E93^{-}*/E93*$^{-}$	
	+/+; en-Gal4>UAS-GFP/UAS-HDAC3 RNAi; E93^{-}*/E93*$^{-}$	
	+/+; en-Gal4>UAS-GFP/UAS-HDAC3 RNAi; UAS-Diap1::E93^{-}*/E93*$^{-}$	
**e**, **h**	*+/+; Gal80ts, en-Gal4>UAS-GFP/+; UAS-P300::E93*$^{-}$*/E93*$^{-}$	18-29°C
	+/+; Gal80ts, en-Gal4>UAS-GFP/+; UAS-P300::UAS-Diap1::E93^{-}*/ E93*$^{-}$	

In **b**, the fat body were dissected from 96 h AEL to WPP at intervals of 2 hours.

In **c**, the salivary gland were dissected from 96 h AEL to 12 h APF at intervals of 4 hours.

In **d** and **g**, the progeny were cultured at 29°C to EW stage before dissection.

In **e** and **h**, the progeny were cultured at 18°C from egg laying until the mid 3rd instar, and then were cultured at 29°C to EW stage before dissection.

Figure S8, related to Figure 4

**b**, **c**	w^{1118}	25°C
**d**	*+/+; tub-Gal4/+;* $E93^-/E93^-$	25°C
	+/+; tub-Gal4/UAS-CtBP RNAi; $E93^-/E93^-$	
	+/+; tub-Gal4/UAS-HDAC3 RNAi; $E93^-/E93^-$	
	+/+; tub-Gal4/UAS-P300 RNAi; $E93^-/E93^-$	
**e**	*upd-Gal4/+; UAS-GFP/+; +/+*	
**f**	*upd-Gal4/+; rpr promoter-GFP/+; +/+*	29°C
	upd-Gal4/+; hid promoter-GFP/+; +/+	
	upd-Gal4/+; grim promoter-GFP/+; +/+	
**g**	*upd-Gal4/+; rpr H3K14ac/H4K8ac promoter-GFP/+;* $E93^-/E93^-$	
	upd-Gal4/+; rpr H3K14ac/H4K8ac promoter-GFP/UAS-CtBP RNAi; $E93^-/E93^-$	
	upd-Gal4/+; rpr H3K14ac/H4K8ac promoter-GFP/UAS-HDAC3 RNAi; $E93^-/E93^-$	
	Gal80ts, upd-Gal4/+; rpr H3K14ac/H4K8ac promoter-GFP/+; UAS-P300::$E93^-/E93^-$	29-25°C
**h**	*upd-Gal4/+; hid H3K14ac/H4K8ac promoter-GFP/+;* $E93^-/E93^-$	29°C
	upd-Gal4/+; hid H3K14ac/H4K8ac promoter-GFP/UAS-CtBP RNAi; $E93^-/E93^-$	
	upd-Gal4/+; hid H3K14ac/H4K8ac promoter-GFP/UAS-HDAC3 RNAi; $E93^-/E93^-$	
	Gal80ts, upd-Gal4/+; hid H3K14ac/H4K8ac promoter-GFP/+; UAS-P300::$E93^-/E93^-$	29-25°C
**i**	*upd-Gal4/+; grim promoter-GFP/+;* $E93^-/E93^-$	29°C
	upd-Gal4/+; grim promoter-GFP/UAS-CtBP RNAi; $E93^-/E93^-$	
	upd-Gal4/+; grim promoter-GFP/UAS-HDAC3 RNAi; $E93^-/E93^-$	
	Gal80ts, upd-Gal4/+; grim promoter-GFP/+; UAS-P300::$E93^-/E93^-$	29-25°C
In **b** and **c**, the whole body of progeny were collected at 96 h AEL and 6 h APF.		

In **d**, the progeny of *CtBP RNAi* and *HDAC3 RNAi* were collected at EW stage; the progeny of *P300 RNAi* were collected at 6 h APF.

In **e**, the progeny were dissected at EW stage.

In **f**, the progeny were cultured at 29°C from egg laying to EW stage before dissection.

In **g-i**, the progeny of control, *CtBP RNAi*, and *HDAC3 RNAi* were cultured at 29°C from egg laying to EW stage before dissection. The progeny of *UAS-P300* were cultured at 29°C after egg laying for 24 h, and then were cultured at 25°C to EW stage before dissection.

**Figure 5**		
**a**	*+/+; ppl-Gal4; +/+*	29°C
	+/+; ppl-Gal4/+; UAS-Tip60 RNAi/+	
	+/+; ppl-Gal4; UAS-Tip60 RNAi::UAS-Diap1/+	
**b**, **c**	*hsFlp/+; UAS-dicer/+; Act5c>CD2>Gal4>UAS-GFP/UAS-Tip60 RNAi*	
	hsFlp/+; UAS-dicer/+; Act5c>CD2>Gal4>UAS-GFP/UAS-Tip60 RNAi::UAS-Diap1	
	hsFlp/+; UAS-p35/UAS-dicer; Act5c>CD2>Gal4>UAS-GFP/UAS-Tip60 RNAi	
**d**	*+/+; ppl-Gal4/+; +/+*	25°C
	+/+; ppl-Gal4/+; UAS-Tip60 RNAi/+	
**f**	w^{1118}	
**g**	*+/+; tub-Gal4/+; +/+*	25°C
	+/+; tub-Gal4/+; UAS-Tip60 RNAi/+	
	+/+; tub-Gal4/UAS-P300 RNAi; +/+	
**h**	*hsFlp/+; Diap1 H3K14ac/H3K27ac/H4K8ac promoter-GFP/+; Act5c>CD2>Gal4>UAS-RFP/UAS-Tip60 RNAi*	
	hsFlp/+; Diap1 H3K14ac/H3K27ac/H4K8ac promoter-GFP/UAS-P300 RNAi; Act5c>CD2>Gal4>UAS-RFP/+	
**i**	*hsFlp/+; Diap1 H3K14ac/H3K27ac/H4K8ac promoter-GFP/+; Act5c>CD2>Gal4>UAS-RFP/UAS-Tip60*	
	hsFlp/+; Diap1 H3K14ac/H3K27ac/H4K8ac promoter-GFP/+;	

	Act5c>CD2>Gal4>UAS-RFP/UAS-P300	

In **a**, the progeny were dissected at EW stage.

In **b**, **c**, **h**, and **i**, the progeny were heat shocked at 37°C for 15 min at 24 h AEL and were then cultured at the indicated temperature to indicated stage before dissection. In **b** and **c**, EW stage; In **h** and **i**, *Tip60-i* and *P300-i* were dissected at 8-12 h APF; *UAS-Tip60* and *UAS-P300* were dissected at EW stage.

In **d**, the fat body of progeny were dissected at 6 h APF.

In **f**, the whole body of progeny were collected at 96 h AEL and 6 h APF.

In **g**, the whole body of progeny were collected at 6 h APF.

**Figure S9, related to Figure 5**		
**a**	*+/+; ppl-Gal4; +/+*	25°C
	+/+; ppl-Gal4/+; UAS-CtBP RNAi/+	
**b**, **d**	w^{1118}	
**e**	*hsFlp/+; UAS-dicer/+; Act5c>CD2>Gal4>UAS-GFP/UAS-Hat1 RNAi*	29°C
	hsFlp/+; UAS-dicer/+; Act5c>CD2>Gal4>UAS-GFP/UAS-gcn5 RNAi	
	hsFlp/+; UAS-dicer/+; Act5c>CD2>Gal4>UAS-GFP/UAS-P300 RNAi	
	hsFlp/+; UAS-dicer/+; Act5c>CD2>Gal4>UAS-GFP/UAS-Taf1 RNAi	
	hsFlp/+; UAS-dicer/+; Act5c>CD2>Gal4>UAS-GFP/UAS-Tip60 RNAi	
	hsFlp/+; UAS-enok RNAi/UAS-dicer; Act5c>CD2>Gal4>UAS-GFP/+	
	hsFlp/+; UAS-dicer/+; Act5c>CD2>Gal4>UAS-GFP/UAS-chm RNAi	
	hsFlp/+; UAS-dicer/+; Act5c>CD2>Gal4>UAS-GFP/UAS-mof RNAi	
	hsFlp/+; UAS-dicer/+; Act5c>CD2>Gal4>UAS-GFP/UAS-Tai RNAi	
	hsFlp/+; UAS-dicer/+; Act5c>CD2>Gal4>UAS-GFP/UAS-clk RNAi	
	hsFlp/+; UAS-dicer/+; Act5c>CD2>Gal4>UAS-GFP/UAS-Atac2 RNAi	

In a, the fat body were dissected at EW, LW, and WPP stage, respectively.

In b and d, the fat body were dissected from 96 h AEL to 8 h APF at intervals of 2 hours.

In e, the progeny were heat shocked at 37°C for 15 min at 24 h AEL and were then cultured at the indicated temperature to EW stage before dissection.

**Figure S10, related to Figure 5**

**a**, **b**	*+/+; en-Gal4>UAS-GFP/+; UAS-Tip60 RNAi/+*	29℃
	+/+; en-Gal4>UAS-GFP/+; UAS-Tip60 RNAi::UAS-Diap1/+	
	+/+; en-Gal4>UAS-GFP/UAS-p35; UAS-Tip60 RNAi/+	
**c**	*hsFlp/+; UAS-dicer/+; Act5c>CD2>Gal4>UAS-GFP/UAS-CtBP RNAi*	29℃
**e**	*+/+; ppl-Gal4; +/+*	25℃
	+/+; ppl-Gal4/+; UAS-Tip60/+	
**f-h**	*+/+; tub-Gal4; +/+*	25℃
	+/+; tub-Gal4/+; UAS-Tip60 RNAi/+	
	+/+; tub-Gal4/UAS-P300 RNAi; +/+	
**i**	*hsFlp/+; Diap1 H3K14ac/H3K27ac/H4K8ac promoter-GFP/UAS-dicer; Act5c>CD2>Gal4>UAS-GFP/UAS-CtBP RNAi*	
	hsFlp/+; Diap1 H3K14ac/H3K27ac/H4K8ac promoter-GFP/UAS-dicer; Act5c>CD2>Gal4>UAS-GFP/UAS-HDAC3 RNAi	
In **a**, **b**, and **e**, the progeny were dissected at EW stage.<br>In **c** and **i**, the progeny were heat shocked at 37°C for 15 min at 24 h AEL and were then cultured at the indicated temperature to EW stage before dissection.<br>In **f-h**, the whole body of progeny were collected at 6 h APF.		

**Figure S11, related to Figure 6**		
**c**	*hsFlp/+; UAS-HomoCtBP/+; Act5c>CD2>Gal4>UAS-GFP/+*	25℃
	hsFlp/+; UAS-HomoHDAC3/+; Act5c>CD2>Gal4>UAS-GFP/+	
	hsFlp/+; UAS-HomoCREBBP/+; Act5c>CD2>Gal4>UAS-GFP/+	
	hsFlp/+; UAS-HomoKAT5/+; Act5c>CD2>Gal4>UAS-GFP/+	
The progeny were heat shocked at 37°C for 15 min at 24 h AEL and were then cultured at 25℃ to the indicated stage before dissection. The progeny of *UAS-HomoCtBP*, *UAS-HomoHDAC3*, and *UAS-HomoKAT5* were dissected at 8-12 h APF, and the progeny of *UAS-HomoCREBBP* were dissected at EW stage.		

**Figure 7**		
**a**	*hsFlp/+; UAS-Ras^{V12}/UAS-dicer; Act5c>CD2>Gal4>UAS-GFP/+*	

	hsFlp/+; UAS-Ras^{V12}*/UAS-dicer; Act5c>CD2>Gal4>UAS-GFP/UAS-CtBP RNAi*	
	hsFlp/+; UAS-Ras^{V12}*/UAS-dicer; Act5c>CD2>Gal4>UAS-GFP/UAS-HDAC3 RNAi*	
	hsFlp/+; UAS-Ras^{V12}*/UAS-dicer; Act5c>CD2>Gal4>UAS-GFP/UAS-Tip60 RNAi*	
	hsFlp/+; UAS-dicer/+; Act5c>CD2>Gal4>UAS-GFP/UAS-Yki^{3SA}	
**b**	*hsFlp/+; UAS-CtBP RNAi/UAS-dicer; Act5c>CD2>Gal4>UAS-GFP/UAS-Yki*^{3SA}	
	hsFlp/+; UAS-HDAC3 RNAi/UAS-dicer; Act5c>CD2>Gal4>UAS-GFP/UAS-Yki^{3SA}	
	hsFlp/+; UAS-Tip60 RNAi/+; Act5c>CD2>Gal4>UAS-GFP/UAS-Yki^{3SA}	
	upd-Gal4>UAS-GFP/+; UAS-Scrib RNAi/UAS-dicer; +/+	
**c**	*upd-Gal4>UAS-GFP/+; UAS-Scrib RNAi/UAS-dicer; UAS-CtBP RNAi/+*	
	upd-Gal4>UAS-GFP/+; UAS-Scrib RNAi/UAS-dicer; UAS-HDAC3 RNAi/+	
	upd-Gal4>UAS-GFP/+; UAS-Scrib RNAi/UAS-dicer; UAS-Tip60 RNAi/+	
	upd-Gal4>UAS-GFP/+; UAS-l(2)gl RNAi/UAS-dicer; +/+	
**d**	*upd-Gal4>UAS-GFP/+; UAS-l(2)gl RNAi/UAS-dicer; UAS-CtBP RNAi/+*	
	upd-Gal4>UAS-GFP/+; UAS-l(2)gl RNAi/UAS-dicer; UAS-HDAC3 RNAi/+	
	upd-Gal4>UAS-GFP/+; UAS-l(2)gl RNAi/UAS-dicer; UAS-Tip60 RNAi/+	
In **a**, the progeny were cultured at 25°C until 2 days AEL, and were heat shocked at 37°C for 5 min, then were cultured at 25°C to EW stage before dissection.<br>In **b**, the progeny were cultured at 25°C until 3 days AEL, and were heat shocked at 37°C for 10 min, then were cultured at 25°C to EW stage before dissection.<br>In **c** and **d**, the progeny were cultured at 25°C to EW stage before dissection.		

**Figure S15, related to Figure 7**		
**a**	See Fig. 7**a**	
**b**	See Fig. 7**b**	
**c**	See Fig. 7**c**	

**d**	See Fig. 7**d**	
In **a-d**, see Fig. 7**a-d**.		

Supplementary Table 4. Information of Recombinant DNA

Recombinant DNA	SOURCE	IDENTIFIER
pIEx4	This paper	N/A
pIEx4-CtBP-V5	This paper	N/A
pIEx4-HDAC3-Flag	This paper	N/A
pAc-sgRNA-Cas9	Addgene	#49330
pAc-gfp-sgRNA-Cas9	This paper	N/A
pAc-P300-sgRNA-Cas9	This paper	N/A
PGL3-basic	Promega	E1751
PGL3-E93B-R1	This paper	N/A
PGL3-E93B-R2	This paper	N/A
PGL3-E93B-R3	This paper	N/A
PGL3-E93B-R4	This paper	N/A
PGL3-E93B-R5	This paper	N/A
pRL-null	Promega	E2271
pRL-Actin3	This paper	N/A
pH-Stinger	Drosophila Genomics Resource Center	DGRC_1018
pH-Stinger-GFP-E93 H3K27ac/H4K8ac enhancer	This paper	N/A
pH-Stinger-GFP-rpr H3K14/H4K8ac promoter	This paper	N/A
pH-Stinger-GFP-hid H3K14/H4K8ac promoter	This paper	N/A
pH-Stinger-GFP-grim promoter	This paper	N/A
pH-Stinger-GFP-Diap1 H3K14ac/H3K27ac/H4K8ac promoter	This paper	N/A
pUAST-attB	Drosophila Genomics Resource Center	DGRC_1419
pUAST-DmCtBP-V5	This paper	N/A
pUAST-DmHDAC3-Flag	This paper	N/A
pUAST-HomoCtBP	This paper	N/A
pUAST-HomoHDAC3	This paper	N/A
pUAST-HomoCREBBP	This paper	N/A
pUAST-HomoKAT5	This paper	N/A
pMD2G	This paper	N/A
psPAX2	Addgene	#12259
PLKO.1-shScramble-GFP	This paper	N/A
PLKO.1-shCtBP-GFP	This paper	N/A
PLKO.1-shHDAC3-GFP	This paper	N/A
PLKO.1-shCREBBP-GFP	This paper	N/A
PLKO.1-shKAT5-GFP	This paper	N/A

Supplementary Table 5. Information of Primers

Category	Name	Sequence
Ectopic expression in Kc cells	*pIEx4-CtBP-V5*	F: ATGGACAAAAATCTGATGATGCCG
		R: CTACGGCGCCTCCGTTGA
	pIEx4-HDAC3-Flag	F: ATGACGGACCGTAGGGTGTCGTACTTCTAC
		R: CTAACTTTCTGCCGAATCGGGCTTGTCTTG
Knock down in Kc cells	*pAc-gfp-sgRNA-Cas9*	F: TTCGAGCTGGACGGCGACGTAAA
		R: AACTTTACGTCGCCGTCCAGCTC
	pAc-P300-sgRNA-Cas9	F: CCATTATTCGATATTGACA
		R: GCTTTGTTCTCGTCGAACTTTTC
Dual luciferase assay	*PGL3-E93B-R1*	F: ACCACTGCCGTTCTCAGGTC
		R: TACCCGCAATTACTGCTGCGCTCTCA
	PGL3-E93B-R2	F: TGAGAGCGCAGCAGTAATTGCGGGTA
		R: GAGGCGCAGCGAAATATGGA
	PGL3-E93B-R3	F: TCCATATTTCGCTGCGCCTC
		R: GCAGTGCTTTCCGTCTGTCA
	PGL3-E93B-R4	F: TGACAGACGGAAAGCACTGC
		R: GAAATTGAGTCGCAGGCCGA
	PGL3-E93B-R5	F: TCGGCCTGCGACTCAATTTC<br>R: TCACGTAGAATCGAGACCGAGG
ChIP-qPCR	E93 H3K27ac/H4K8ac enhancer in R1	F: CCAGTGCGAATTCGGTTGTC
		R: GTATTCAGTCGGTCGGTCGG
	E93 gene locus in R2	F: CCAAGAACAGCGTGGGATAA
		R: AGCCTTAGCACTCCCCTTTA
	E93 H3K27ac/H4K8ac basic promoter in R3	F: AATGCTGGCTTGCTTGTGTG
		R: CTCGAGCCGACGGACAATAA
	rpr H3K14ac/H4K8ac promoter	F: GAAACGATTGTCATGCCCCG
		R: GGCTGCTCATTTTGGCCTTC
	hid H3K14ac/H4K8ac promoter	F: TCTCCCTCCGCTCTCTTGTT
		R: AGTATATCCGACTGCGCTGC
	grim promoter	F: TTCCGCTCTCATTTCCCGAG
		R: AGACACGGCTGCCTTTGTTA
	Diap1 H3K14ac promoter in R1	F: GGTCGTTGTCGTTGTGTTGT
		R: CGATGAAGAGGGAAGAGCGG
	Diap1 H3K27ac/H4K8ac promoter in R2	F: ACCGCCAGGCTACCAATATG
		R: TCGACTCTGGCTGTAGTTGC
		F: TGACGGCCTAGCGTAGACTT

	Diap1 H4K8ac promoter in R3	R: GTGCTTGGCTCGCTGATGTA
pH-stinger-GFP for transgenic flies	*pH-stinger-GFP-E93 H3K27ac/H4K8ac enhancer*	F: GCCGGCTATAACTGGCTCAC
		R: GAGGCGCAGCGAAATATGGA
	pH-stinger-GFP-rpr H3K14/H4K8ac promoter	F: TAGGCCTTCGAACACGTCGAT
		R: GAATGCCACTGCCATTGTTGGT
	pH-stinger-GFP-hid H3K14/H4K8ac promoter	F: CGTTTTGTGCAAGAGTGTGCTTGAATTTCG
		R: GCACTGGGGCCTCAAACTACGGCAACAGAG
	pH-stinger-GFP-grim promoter	F: CACCTCATTTTCCGCACACCATTTATAAGC
		R: CTTGCTGCAGTCACATTTATAAAATAGCTC
	pH-stinger-GFP-Diap1 H3K14ac/H3K27ac/ H4K8ac promoter	F: ATTATATCCACTAACGAGGGGAC
		R: TAAACCGTGAGCAGCGCCTTC
pUAST for transgenic flies	*pUAST-DmCtBP-V5*	F: ATGGACAAAAATCTGATGATGCCG
		R: CTACGGCGCCTCCGTTGACTCG
	pUAST-DmHDAC3-Flag	F: ATGACGGACCGTAGGGTGTCGT
		R: CTAACTTTCTGCCGAATCGGGC
	pUAST-HomoCtBP	F: ATGGGCAGCTCGCACTTGCTCAAC
		R: TTACAACTGGTCACTGGCGTGG
	pUAST-HomoHDAC3	F: ATGGCCAAGACCGTGGCCTATTTC
		R: TTAAATCTCCACATCGCTTTCC
	pUAST-HomoCREBBP	F: ATGGCTGAGAACTTGCTGGACG
		R: CTACAAGCCCTCCACAAACTTCTC
	pUAST-HomoKAT5	F: ATGGCGGAGGTGGGGGAGATAAT
		R: TTACCACTTCCCCCTCTTGCT
shRNA in mammals	*Scramble*	CCGG-*CCTAAGGTTAAGTCGCCCTCG*-CTCGAG-*CGAGGGCGACTTAACCTTAGG*-TTTTTTT
	CtBP	CCGG-*AGTCGGAACCCTTCAGCTTTA*-CTCGAG-*TAAAGCTGAAGGGTTCCGAC*-TTTTTTT
	HDAC3	CCGG-*GATCTGTGATATTGCCATTAA*-CTCGAG-*TTAATGGCAATATCACAGATC*-TTTTTT
	CREBBP	CCGG-*GCTATCAGAATAGGTATCATT*-CTCGAG-*AATGATACCTATTCTGATAGC*-TTTTTT
	KAT5	CCGG-*TCGAATTGTTTGGGCACTGAT*-CTCGAG-*ATCAGTGCCCAAACAATTCGA*-TTTTTT
q-PCR in *Drosophila*	*rp49*	F: GACAGTATCTGATGCCCAACA
		R: CTTCTTGGAGGAGACGCCGT
	E93B	F: CCTTCGTCTCCAAGTCGTGT
		R: TGCTATTCGATGCTCCATCT

	rpr	F: GAGTCGCCTGATCGGGTATG
		R: CGCCAGCAACAAAGAACTAAC
	hid	F: CACTTGGGACGAGTTTGG
		R: GGCTGGCTATCGGTATGG
	grim	F: GTGCCAATATTTCCGTGCCG
		R: ATCCCAGCATCCAAACTCCG
	Diap1	F: AAATGCTTTTTCTGCGGCGT
		R: CTCATCTCCAGCGTCGAGTC
	CtBP	F: CAACTGGCTGCACCTGAGAG
		R: TCCTCCTCCTCCTCCTCCTC
	HDAC3	F: GGACTGCATCACGCCAAGAA
		R: GAGCACCCGAGGATGGTACT
	P300	F: AAGTAAGCGGCGATGGCAAA
		R: CGCCGAATGCAGACGGATAA
	Tip60	F: TCACCCGCATGAAGAACGTG
		R: ACAGCTCTTGCGGATAGGGT
	Rpd3	F: GCTGACTGGCGATCGGTTAG
		R: TCACGAACTCCACGCACTTG
	HDAC4	F: GCACGATAGCCACCAGTACG
		R: GGTCCCGCAGCTTAATACCG
	HDAC6	F: GCTGACCTGTCTGTGTGGTG
		R: GTCGCATAGCAGCCATCCTC
	Sir2	F: ACGAGCAACGAGGATGAGGA
		R: CGGCACACGACCTGTGTAAA
	Sirt2	F: CGCCGGCCCACTACTTTATC
		R: AGTAAGCCGGTCTAGCGTGT
	Sirt4	F: GTGGAGGTTCATGGCAGTGG
		R: TAGCGACGCCAGTATGCTCT
	Sirt6	F: GGACACGTTGTCCTCCACAC
		R: CTTCTCGCCCTTCTCCTCCA
	Sirt7	F: CTTCTCGCCCTTCTCCTCCA
		R: GGCCCGTTTCTTGTGTTTGC
	Sin3A	F: CCGAAGGCAAGCCAAAGGAT
		R: CTGCTCCTGCAGTGTCTTGG
	Hat1	F: GCAGTATGCCACAGCTGGTT
		R: CTGGCTTATCCTGGGCCTCT
	gcn5	F: CACCCACGAGGATGCCTCTA
		R: CAGGAGCGTTCGTCCAAACA
	Taf1	F: AACCCATCTCTGGCTGACGA
		R: GCTTGCCATCATCACCACCA
	enok	F: CACCATCCAATTCCGCCCAA
		R: TACCTGCGTTTGCAGCATGT

	chm	F: GAATTCTGCCTGCGCTACCA
		R: ACCTGTAGTTTGCCCTTGCG
	mof	F: ACCATGGAGCACGACAACAC
		R: CCATCCTCGCGCCGAATAAA
	Elp3	F: ACATTCCCATGCCGCTTGTT
		R: GCCAACTTCGCGGGTTCTAA
	tai	F: TGCCTATCCGGGAATGCTCA
		R: TCGCTGGAAACCCTGATTGC
	clk	F: CAGATGGCCACCGAATCGAG
		R: AGATTGCTGCCGGAAGTGTG
	Atac2	F: CGTTGCTTCACGCACCTTTG
		R: CGGCTGCAGGTTCTTCTGTT
	EcR-B1	F: CATAGGGGAAATGGGGAAGTTC
		R: TAACTGCATTTTACGCTTGTCC
	USP	F: CTACCAGAAGTGCCTAACCTG
		R: CATGAAGTCATCAGAACCGTTG
	E75	F: CCTCAAGCAGCGCGAGTT
		R: GCGATTTCCTTGTGGGTCT
	Br-C	F: CTCAACACGCACACCCAAT
		R: GCTGAAGAGGGTCGAGGAG
	Hr3	F: TGGACCGTGTTAATCGCAAC
		R: ACCTCGTCCTCGACCTTCTC
	Ftz-f1	F: TGCAGAACAAGAAGGTCTACAC
		R: TTTGTACATGGGTCCGAATTTG
	Met	F: AAACATATGTACGTTGCGTGAG
		R: GTTTTCAAGGTCTCGCTTATGC
	Gce	F: CTGTATGTGAACATCAAGAGCG
		R: ACATCGATGAAACCCTTCAGTC
	Kr-h1	F: GAACATATTCACGGAAATCGGG
		R: CAACCTTCGCGCTTTGATATTA
	Atg1	F: GATCGCAGCCAATTAGCGTAAA
		R: CGGTGAGATCGAGTTAATGTCA
	Atg5	F: GCTACTTGAATCGCACTACATG
		R: GATTGTGGTCCTTCTTTTGCAT
	Atg6	F: AGGAACAATCGCTAAATGATGC
		R: ATGTTGAATATGTTGGTGTCGC
	Atg8	F: CCCAAAAGCAAACGAAGTGATA
		R: CTTCTTCTTGTCCAAATCACCG
	dronc	F: CGCCACTGGACATTTTATCATT
		R: AAACATTTTATAGCACGCGAGC
	drice	F: TGTCGCATTTACTCGTCTGTAT
		R: GCCGGAGTAAAAACCTCTTAGA

	Idh3b	F: CATCGCAATCAAAAATTTCGCG
		R: CATCGACATTTTGCTATTCGGT
q-PCR in mammals	*GAPDH*	R: CGGATTTGGTCGTATTGGGC
		F: GTAGTTGAGGTCAATGAAGGGGTC
	CtBP	F: GTTCAAAGCCCTCCGCATCA
		R: GCCGGTACAGGTTCAGGATG
	HDAC3	F: GCGATGACTGCCCAGTGTTT
		R: TTCTTGGCATGGTGCAGACC
	CREBBP	F: CTGATGAACGATGGCTCCAA
		R: GAAGATGGCTTGGACGAGTT
	KAT5	F: GGCCACAGATCACCATCAAT
		R: GGATGTACTGGCCCTTGTAG
	HtrA2	F: GGACCGGCACCCTTTCTTG
		R: TGGGCGTTGGTGACAATGAG
	Smac	F: CAAGCTGGCAGAAGCACAGA
		R: GTGTGCTCAGGCCCTCAATC
	Bad	F: AGTCGCCACAGCTCCTA
		R: GGCGAGGAAGTCCCTTCTTA
	Bak	F: GTGAAGGCTCTCACCCATC
		R: CCCACTTAGAACCCTCCAGA
	Bax	F: CCCTTTTGCTTCAGGGTTTCA
		R: CTGCAGCTCCATGTTACTGT
	Bid	F: CCTTGCTCCGTGATGTCTTT
		R: TAGCCAGTCACACTTCTGGA
	Bim	F: ATCTCAGTGCAATGGCTTCC
		R: CAGGCGGACAATGTAACGTA
	Puma	F: GCAGGGCAGGAAGTAACAAT
		R: TCCCTGGGGCCACAAAT
	Noxa	F: GCTGGAAGTCGAGTGTGCTA
		R: GAAACGTGCACCTCCTGAGA
	XIAP	F: GGTGACCAAGTGCAGTGCTT
		R: AGTGTCGCCTGTGTTCTGAC
	Bcl-2	F: GGAGGATTGTGGCCTTCTTT
		R: GTTCCACAAAGGCATCCCAG
	Bcl-xL	F: GGGGCTCGCAGAATCTTAT
		R: TCACTGAGTCTCGTCTCTGG

Supplementary Table 6. Information of Antibodies

Antibodies	SOURCE	IDENTIFIER
Active Caspase-3	abcam	ab32042
Cytochrome C	abcam	ab133504
V5-Mouse	CST	#80076
V5-Rabbit	abcam	ab9116
Flag-Mouse	Merck	F1084
Flag-Rabbit	Merck	F7425
EcR-B1	DSHB	AD4.4
RNA polymerase II	abcam	ab300575
CtBP	abclonal	A1707
HDAC3	abclonal	A2139
Mouse IgG	Merck	12-371
Rabbit IgG	Merck	12-370
Goat anti-Rabbit IgG (H+L)	Invitrogen	31210
H2AK5ac	abclonal	A15620
H3K14ac	abclonal	A7254
H3K18ac	abclonal	A7257
H3K27ac	abclonal	A7253
H4K5ac	abclonal	A15233
H4K8ac	abclonal	A7258
H4K12ac	abclonal	A14227
H4K16ac	abcam	ab109463
Caspase-3	Beyotime	AC030
Caspase-9	Beyotime	AC062
β-actin	Beyotime	AF0003
HRP-labeled Goat anti-Mouse IgG (H+L)	Beyotime	A0216
HRP-labeled Goat anti-Rabbit IgG (H+L)	Beyotime	A0208
Alexa Fluor™ 594 Goat anti-Mouse IgG	Invitrogen	A-11032
Alexa Fluor™ 594 Goat anti-Rabbit IgG	Invitrogen	A-11037
Alexa Fluor™ 488 Goat anti-Mouse IgG	Invitrogen	A-11029
Alexa Fluor™ 488 Goat anti-Rabbit IgG	Invitrogen	A-11034
Alexa Fluor™ 647 Goat anti-Mouse IgG	Invitrogen	A-32728
Alexa Fluor™ 647 Goat anti-Rabbit IgG	Invitrogen	A-21245
