# Supplementary material for: Histone acetylation homeodynamics navigates cell survival and apoptosis
Source: Nat Commun. 2025 Dec 12;16:11358. doi: 10.1038/s41467-025-66405-4 (PMC12728225; doi:10.1038/s41467-025-66405-4)
Supplement: Supplementary file 2 — Reporting Summary [file 41467_2025_66405_MOESM2_ESM.pdf]

nature portfolio

Corresponding author(s): Sheng Li

Last updated by author(s): Oct 19, 2025

Reporting Summary

Nature Portfolio wishes to improve the reproducibility of the work that we publish. This form provides structure for consistency and transparency in reporting. For further information on Nature Portfolio policies, see our Editorial Policies and the Editorial Policy Checklist.

Statistics

For all statistical analyses, confirm that the following items are present in the figure legend, table legend, main text, or Methods section.

n/a	Confirmed	
☐	☒	The exact sample size (*n*) for each experimental group/condition, given as a discrete number and unit of measurement
☐	☒	A statement on whether measurements were taken from distinct samples or whether the same sample was measured repeatedly
☐	☒	The statistical test(s) used AND whether they are one- or two-sided<br>*Only common tests should be described solely by name; describe more complex techniques in the Methods section.*
☒	☐	A description of all covariates tested
☒	☐	A description of any assumptions or corrections, such as tests of normality and adjustment for multiple comparisons
☐	☒	A full description of the statistical parameters including central tendency (e.g. means) or other basic estimates (e.g. regression coefficient) AND variation (e.g. standard deviation) or associated estimates of uncertainty (e.g. confidence intervals)
☐	☒	For null hypothesis testing, the test statistic (e.g. *F*, *t*, *r*) with confidence intervals, effect sizes, degrees of freedom and *P* value noted<br>*Give P values as exact values whenever suitable.*
☒	☐	For Bayesian analysis, information on the choice of priors and Markov chain Monte Carlo settings
☒	☐	For hierarchical and complex designs, identification of the appropriate level for tests and full reporting of outcomes
☒	☐	Estimates of effect sizes (e.g. Cohen's *d*, Pearson's *r*), indicating how they were calculated

Our web collection on statistics for biologists contains articles on many of the points above.

Software and code

Policy information about availability of computer code

Data collection	Commercial softwares licensed by microscopy companies were utilized: FV31S-SW (for Olympus FV3000).
Data analysis	For RNA-Seq analysis, differential expression analysis of the two conditions/groups was performed via DESeq2 (Love MI, Huber W, Anders S. Moderated estimation of fold change and dispersion for RNA-seq data with DESeq2. Genome biology 15, (2014).). For CUT&Tag analysis, filtering tools (FastQC and Trimmomatic) was used to remove the interference information, containing adapter sequence, low-quality bases and undetected bases in paired-end raw data (Bolger AM, Lohse M, Usadel B. Trimmomatic: a flexible trimmer for Illumina sequence data. Bioinformatics 30, 2114-2120 (2014)). Bowtie2 was used to map clean reads to the Drosophila genome (Langmead B, Salzberg SL. Fast gapped-read alignment with Bowtie 2. Nature Methods 9, 357-359 (2012)). Samtools and Picard were used to screen out the low-quality mapping, PCR redundancy and organelle alignment (Danecek P, et al. Twelve years of SAMtools and BCFtools. GigaScience 10, (2021)). MACS2 software was used to perform Peak Calling (Zhang Y, et al. Model-based Analysis of ChIP-Seq (MACS). Genome biology 9, (2008)). Deeptools was used for peak normalization (Ramírez F, et al. deepTools2: a next generation web server for deep-sequencing data analysis. Nucleic Acids Res 44, W160-W165 (2016)). The IGV (Integrative Genomic Viewer) was used to visualize enrichment regions or the called peaks on genome (Robinson JT, et al. Integrative genomics viewer. Nat Biotechnol 29, 24-26 (2011)).

For manuscripts utilizing custom algorithms or software that are central to the research but not yet described in published literature, software must be made available to editors and reviewers. We strongly encourage code deposition in a community repository (e.g. GitHub). See the Nature Portfolio guidelines for submitting code & software for further information.

Data

Policy information about availability of data

All manuscripts must include a data availability statement. This statement should provide the following information, where applicable:

- Accession codes, unique identifiers, or web links for publicly available datasets
- A description of any restrictions on data availability
- For clinical datasets or third party data, please ensure that the statement adheres to our policy

Source data are provided with this paper. All the raw sequencing data are deposited in SRA. RNA-seq of CtBP-i in Drosophila fat body, SRA: PRJNA1170300. CUT&Tag of H3K14ac/H3K27ac/H4K8ac in Drosophila whole body, SRA: PRJNA1171455. CUT&Tag of H3K14ac/H3K27ac/H4K8ac in A549 cells, SRA: PRJNA1171109. All other relevant data supporting the key findings of this study are available within the article and its supplementary information files. Any additional information is available upon request to the corresponding author (Sheng Li, lisheng@scnu.edu.cn).

Research involving human participants, their data, or biological material

Policy information about studies with human participants or human data. See also policy information about sex, gender (identity/presentation), and sexual orientation and race, ethnicity and racism.

Reporting on sex and gender	Not relevant for this study.
Reporting on race, ethnicity, or other socially relevant groupings	Not relevant for this study.
Population characteristics	Not relevant for this study.
Recruitment	Not relevant for this study.
Ethics oversight	Not relevant for this study.

Note that full information on the approval of the study protocol must also be provided in the manuscript.

Field-specific reporting

Please select the one below that is the best fit for your research. If you are not sure, read the appropriate sections before making your selection.

[x] Life sciences [ ] Behavioural & social sciences [ ] Ecological, evolutionary & environmental sciences

For a reference copy of the document with all sections, see nature.com/documents/nr-reporting-summary-flat.pdf

Life sciences study design

All studies must disclose on these points even when the disclosure is negative.

Sample size	For CUT&Tag experiments in A549 cells, 1× 10 5 cells were used each group. For CUT&Tag experiments in Drosophila whole body experiments, twenty animals were used each developmental stage. For Annexin V-phycoerythrin (PE) staining, 6 × 10 4 cells were used each group. For the tumorigenesis assay, viable A549 cells from each group (2 × 10 6 cells/100 µl of PBS per mouse) were subcutaneously injected. All sample sizes were chosen to ensure the reproducibility of the results.
Data exclusions	No data were excluded from the analyses.
Replication	For RNA-Seq samples, there are three biological replicates each for the control and interference groups. For CUT&Tag samples, there are two biological replicates for materials from different developmental stages. For q-PCR, western blotting, and ChIP-qPCR samples, there are three biological replicates for each treatment group. For the statistical graphs of immunofluorescence, the protein fluorescence intensities were quantified from four independent images for each treatment group. For the mouse tumor formation samples, six mice each from the control and experimental groups were used for the statistical analysis of tumor areas. For the statistical analysis of tumor area or size in Drosophila wing discs, the details are specified in the respective figure legends. All information regarding replication is included in the figure legends. All attempts at replication were successful.
Randomization	Randomization was not necessary for this study. All experiments were carried out under controlled experimental conditions.
Blinding	Blinding was not possible as experimental conditions were mostly evident from the image data.

Reporting for specific materials, systems and methods

We require information from authors about some types of materials, experimental systems and methods used in many studies. Here, indicate whether each material, system or method listed is relevant to your study. If you are not sure if a list item applies to your research, read the appropriate section before selecting a response.

Materials & experimental systems

n/a	Involved in the study
☐	☒ Antibodies
☐	☒ Eukaryotic cell lines
☒	☐ Palaeontology and archaeology
☐	☒ Animals and other organisms
☒	☐ Clinical data
☒	☐ Dual use research of concern
☒	☐ Plants

Methods

n/a	Involved in the study
☐	☒ ChIP-seq
☐	☒ Flow cytometry
☒	☐ MRI-based neuroimaging

Antibodies

Antibodies used

The full list is also available in Supplementary Information, Supplementary Table 6.
Active Caspase-3 abcam ab32042
Cytochrome C abcam ab133504
V5-Mouse Cell Signaling #80076
V5-Rabbit abcam ab9116
Flag-Mouse Merck F1084
Flag-Rabbit Merck F7425
EcR-B1 DSHB AD4.4
RNA polymerase II abcam ab300575
CtBP abclonal A1707
HDAC3 abclonal A2139
Mouse IgG Merck 12-371
Rabbit IgG Merck 12-370
Goat anti-Rabbit IgG (H+L) Invitrogen 31210
H2AK5ac abclonal A15620
H3K14ac abclonal A7254
H3K18ac abclonal A7257
H3K27ac abclonal A7253
H4K5ac abclonal A15233
H4K8ac abclonal A7258
H4K12ac abclonal A14227
H4K16ac abcam ab109463
Caspase-3 Beyotime AC030
Caspase-9 Beyotime AC062
β-actin Beyotime AF0003
HRP-labeled Goat anti-Mouse IgG (H+L) Beyotime A0216
HRP-labeled Goat anti-Rabbit IgG (H+L) Beyotime A0208
Alexa Fluor™ 594 Goat anti-Mouse IgG Invitrogen A-11032
Alexa Fluor™ 594 Goat anti-Rabbit IgG Invitrogen A-11037
Alexa Fluor™ 488 Goat anti-Mouse IgG Invitrogen A-11029
Alexa Fluor™ 488 Goat anti-Rabbit IgG Invitrogen A-11034
Alexa Fluor™ 647 Goat anti-Mouse IgG Invitrogen A-32728
Alexa Fluor™ 647 Goat anti-Rabbit IgG Invitrogen A-21245

Validation

All antibodies used are from commercial sources as described. The validation and references for each are publicly available on the respective vendor websites that can reached via the catalog numbers listed above.

Eukaryotic cell lines

Policy information about cell lines and Sex and Gender in Research

Cell line source(s)

Drosophila Kc cells were obtained from the Drosophila Genomics Resource Center (DGRC), HEK293T and the human lung cancer cell line A549 cells were obtained from the American Type Culture Collection (ATCC).

Authentication

Drosophila Kc cells were maintained in Schneider's Drosophila Medium containing L-glutamine (Sigma) supplemented with 5% fetal bovine serum (FBS; Gibco) and 1% penicillin-streptomycin (Gibco) at 27°C. HEK293T and the human lung cancer cell line A549 were maintained in DMEM medium (Gibco) supplemented with 10% FBS (Gibco), 1% penicillin-streptomycin, and 1% GlutaMAX (Gibco), and cultured at 37°C in a humidified incubator with 5% CO2. All the cells were authenticated by PCR assays with species-specific primers.

Mycoplasma contamination

All the cell lines were tested for mycoplasma contamination, and the experiments were conducted in mycoplasma-negative cells.

Commonly misidentified lines (See ICLAC register)	No commonly misidentified cell lines were used.

Animals and other research organisms

Policy information about studies involving animals; ARRIVE guidelines recommended for reporting animal research, and Sex and Gender in Research

Laboratory animals	BALB/c nude mice was used for xenograft model.
Wild animals	The study did not involve wild animals.
Reporting on sex	For the xenograft model, male BALB/c nude mice (4-6 weeks old) were purchased from GemPharmatech (Nanjing, China) and maintained in an SPF environment.
Field-collected samples	The study did not involve samples collected from the field.
Ethics oversight	The experiments involving mice were performed in strict accordance with the recommendations in the Guide for the Care and Use of Laboratory Animals of the National Institutes of Health. The protocol was approved by the Committee on the Ethics of Animal Experiments of South China Agricultural University.

Note that full information on the approval of the study protocol must also be provided in the manuscript.

Plants

Seed stocks	Not relevant for this study.
Novel plant genotypes	Not relevant for this study.
Authentication	Not relevant for this study.

ChIP-seq

Data deposition

- [x] Confirm that both raw and final processed data have been deposited in a public database such as GEO.
- [ ] Confirm that you have deposited or provided access to graph files (e.g. BED files) for the called peaks.

Data access links<br>*May remain private before publication.*	CUT&Tag of H3K14ac/H3K27ac/H4K8ac in Drosophila whole body: https://submit.ncbi.nlm.nih.gov/subs/sra/SUB14781063/overview. CUT&Tag of H3K14ac/H3K27ac/H4K8ac in A549 cells: https://submit.ncbi.nlm.nih.gov/subs/sra/SUB14778656/overview.
Files in database submission	CUT&Tag of H3K14ac/H3K27ac/H4K8ac in Drosophila whole body, SRA: PRJNA1171455. CUT&Tag of H3K14ac/H3K27ac/H4K8ac in A549 cells, SRA: PRJNA1171109.
Genome browser session (e.g. UCSC)	Not relevant for this study.

Methodology

Replicates	Each CUT&Tag experiment was repeated twice.
Sequencing depth	All CUT&Tag sequencing depth is 6 Gigabases. Total number of reads is from 1 x 10 8 to 2 x 10 8 and uniquely mapped reads is from 1 x 10 7 to 1 x 10 8. The length of reads are 100-300 base pair and the sequences are paired-end.
Antibodies	H3K14ac, Abclonal, cat. no. A7254; H3K27ac, Abclonal, cat. no. A7253; H4K8ac, Abclonal, cat. no. A7258.
Peak calling parameters	MACS2 software with parameters callpeak -t <bam-file-of-mapped-fragments> --keep-dup all --extsize 200 --nomodel -q 0.05 -B
Data quality	We chose MACS2 software based on statistical methods to perform Peak Calling, where the peaks called are significantly enriched regions from retained valid pairs data (q-value <= 0.05). For the sequencing experiments with biological replication, we compared the two of replicate samples to show the overlap between peaks and further evaluate the consistency of peak enrichment multiples. The result of the latter was obtained by a more stringent approach called IDR (Irreproduciable Discovery Rate), which is based on the presence or absence of Overlap Peak, considering the consistency of the order of peak enrichment multiples between the two sets of data.

Software | Please see above data analysis of software and code.

Flow Cytometry

Plots

Confirm that:

- [x] The axis labels state the marker and fluorochrome used (e.g. CD4-FITC).
- [x] The axis scales are clearly visible. Include numbers along axes only for bottom left plot of group (a 'group' is an analysis of identical markers).
- [x] All plots are contour plots with outliers or pseudocolor plots.
- [x] A numerical value for number of cells or percentage (with statistics) is provided.

Methodology

Sample preparation

Aspirate the cell culture medium and wash the A549 cells once with PBS, add an appropriate amount of trypsin solution (EDTA is optimal) and incubate at room temperature until the cells can be released by gently pipetting up and down. Remove and discard the trypsin solution. Add the cell culture medium and blow down gently to release the cells.

Instrument

The fluorescence intensity was evaluated with a BD Fortessa flow cytometer (BD Biosciences, USA).

Software

The data were analyzed with FlowJo V10 software.

Cell population abundance

For Annexin V-phycoerythrin (PE) staining, 6 × 10 4 cells were used each group. Cells were collected by centrifuge at 1000×g for 5 minutes. Discard the supernatant and resuspend cells in PBS gently. Count the number of cells. Take 6 × 10 4 suspension cells, centrifuge at 1000×g for 5 minutes, discard the supernatant, and gently resuspend the cells in 195 µl of Annexin V-PE Binding Buffer. Add 5µl of Annexin V-PE and mix gently. Incubate at room temperature (20-25°C) in the dark for 10-20 minutes and then place on ice. Gently mix the cells 2-3 times during the incubation to improve the staining results. After staining, the cells were examined immediately by flow cytometry.

Gating strategy

The cells were gated based on side scatter (SSC) and Annexin V-PE signals.

- [x] Tick this box to confirm that a figure exemplifying the gating strategy is provided in the Supplementary Information.
